# Nuclear farnesoid X receptor protects against bone loss by driving osteoblast differentiation through stabilizing RUNX2

**DOI:** 10.1038/s41413-024-00394-w

**Published:** 2025-01-30

**Authors:** Qi Dong, Haoyuan Fu, Wenxiao Li, Xinyu Ji, Yingchao Yin, Yiran Zhang, Yanbo Zhu, Guoqiang Li, Huiyang Jia, Heng Zhang, Haofei Wang, Jinglue Hu, Ganggang Wang, Zhihao Wu, Yingze Zhang, Sujuan Xu, Zhiyong Hou

**Affiliations:** 1 Department of Orthopedic Surgery, Third Hospital of Hebei Medical University, Shijiazhuang, Hebei China; 2 Orthopaedic Research Institute of Hebei Province, Third Hospital of Hebei Medical University, Shijiazhuang, Hebei China; 3 Department of Cardiology, Third Hospital of Hebei Medical University, Shijiazhuang, Hebei China; 4 School of Medicine, Nankai University, Tianjin, China; 5 Hebei Food Safety Key Laboratory, Key Laboratory of Special Food Supervision Technology for State Market Regulation, Hebei Engineering Research Center for Special Food Safety and Health, Hebei Food Inspection and Research Institute, Shijiazhuang, Hebei China; 6 Pudong Hospital, Fudan University, Shanghai, China; 7 School of Preclinical Medicine, Wannan Medical College, Wuhu, Anhui China; 8 Hebei Key Laboratory for Diabetic Kidney Disease, Third Hospital of Hebei Medical University, Shijiazhuang, Hebei China; 9 Department of Nephrology, Third Hospital of Hebei Medical University, Shijiazhuang, Hebei China

**Keywords:** Bone, Pathogenesis

## Abstract

The delicate balance between bone formation by osteoblasts and bone resorption by osteoclasts maintains bone homeostasis. Nuclear receptors (NRs) are now understood to be crucial in bone physiology and pathology. However, the function of the Farnesoid X receptor (FXR), a member of the NR family, in regulating bone homeostasis remains incompletely understood. In this study, in vitro and in vivo models revealed delayed bone development and an osteoporosis phenotype in mice lacking FXR in bone marrow mesenchymal stem cells (BMSCs) and osteoblasts due to impaired osteoblast differentiation. Mechanistically, FXR could stabilize RUNX2 by inhibiting Thoc6-mediated ubiquitination, thereby promoting osteogenic activity in BMSCs. Moreover, activated FXR could directly bind to the Thoc6 promoter, suppressing its expression. The interaction between RUNX2 and Thoc6 was mediated by the Runt domain of RUNX2 and the WD repeat of Thoc6. Additionally, Obeticholic acid (OCA), an orally available FXR agonist, could ameliorate bone loss in an ovariectomy (OVX)-induced osteoporotic mouse model. Taken together, our findings suggest that FXR plays pivotal roles in osteoblast differentiation by regulating RUNX2 stability and that targeting FXR may be a promising therapeutic approach for osteoporosis.

## Introduction

Bone remodeling is essential for maintaining bone homeostasis. It involves two continuous processes: bone formation and resorption. The delicate balance between these processes is crucial for preserving bone mass and systemic mineral homeostasis throughout life.^1^ During this process, the original bone is removed by osteoclasts, which are specialized multinucleated cells derived from hematopoietic precursors,^2^ and then rebuilt by osteoblasts derived from bone marrow mesenchymal stem cells (BMSCs).^3^ An imbalance in this process can lead to various bone diseases, including osteoporosis, osteoarthritis, and osteopetrosis. Of these, osteoporosis is the most prevalent disabling disorder particularly affecting elderly individuals, especially postmenopausal women.^4^ Consequently, identifying mechanisms that can stimulate osteoblast differentiation and/or inhibit osteoclast genesis is crucial for developing improved treatments for osteoporosis.

Nuclear receptors (NRs) are a family of ligand-activated transcription factors that play critical roles in bone physiology and pathophysiology.^5,6^ The farnesoid X receptor (FXR, also known as NR1H4) is an adopted member of the nuclear receptor superfamily activated by endogenous ligand bile acids. It functions as an intracellular sensor involved in lipid, bile acids, glucose, cholesterol metabolism, and drug clearance.^7,8^ Except for the liver, small intestine and kidney, FXR is highly expressed in bone.^9,10^ Bone tissue contains bile acid, which is accumulated from serum and can be released into the bone microenvironment in large quantities.^9^ Previous studies have suggested that FXR is essential in regulating bone mass.^9^ However, the underlying mechanisms by which FXR influences bone homeostasis remain to be fully elucidated.

Runt-related transcription factor 2 (RUNX2) is a key transcription factor controlling skeletal development and morphogenesis in vertebrates.^11–13^ Recent studies have demonstrated that RUNX2 haploinsufficiency results in cleidocranial dysplasia (CCD) in humans and mice, which is characterized by incomplete fontanelle closure, hypoplastic clavicles, short stature, and skeletal dysplasia.^12^ Consequently, regulating RUNX2 expression and transcriptional activity is critical for osteoblast differentiation and normal bone formation. Although some studies have explored the regulation of RUNX2 activity and stability though ubiquitination,^14–19^ phosphorylation,^14,20,21^ acetylation^22,23^ or other post-translational modification of proteins,^24,25^ the specific post-translational mechanisms that control RUNX2 to induce BMSCs to differentiate into the osteoblast lineage and subsequently sustain osteoblast differentiation remain to be fully elucidated.

Herein, we investigated the role of FXR in osteoblast differentiation. Our findings demonstrated that FXR deficiency in BMSCs or osteoblasts, but not in mature osteoclast, led to osteoporosis. Additionally, FXR deficiency could significantly inhibits osteoblast differentiation and calcification in vitro. We further elucidated that FXR could reduce the ubiquitination of RUNX2 by suppressing Thoc6 expression. Finally, obeticholic acid (OCA), an orally bioavailable FXR agonist, attenuates bone loss in an ovariectomy (OVX)-induced osteoporotic mouse model. Collectively, our results indicate the positive role of FXR in osteoblast-mediated bone formation through the maintenance of RUNX2 stability. These findings may expand the potential clinical applications of FXR and offer a novel therapeutic strategy for the treatment of osteoporosis.

## Results

### The expression of FXR in osteogenic differentiation and bone formation

**Fig. 1:**
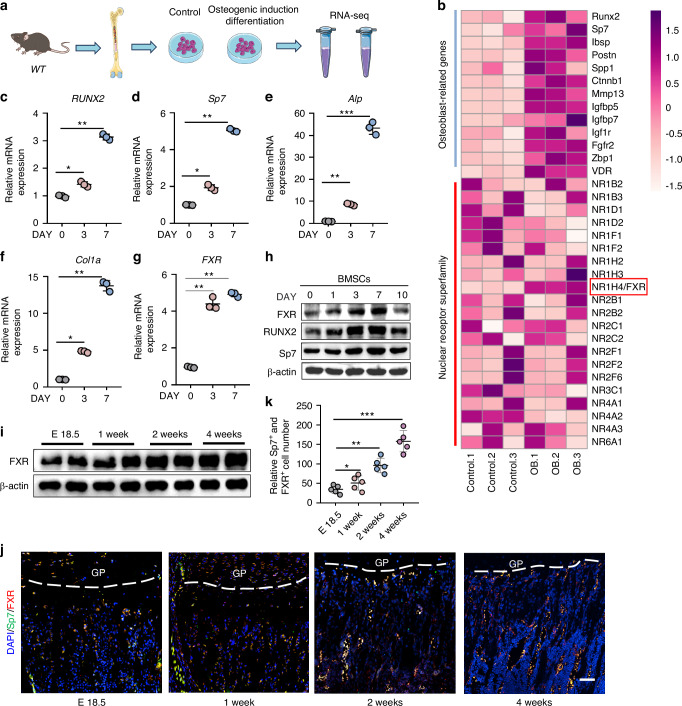
The expression of FXR in osteogenic differentiation and bone formation. **a** Schematic graph of the RNA-seq strategy to identify Nuclear Receptor Superfamily genes which may regulated osteogenic differentiation. Total RNA was isolated from BMSCs, which was cultured from 4-week male WT mice, after 4 days of osteoblastic differentiation followed by RNA sequencing analysis. Kolmogorov–Smirnov (K–S) test was used for testing the correlation between the Control and osteoblast differentiation set. **b** Heatmap of Osteoblast-related genes and Nuclear Receptor Superfamily mRNA expression in BMSCs after 4 days of osteoblastic differentiation. OB Osteoblast differentiation. **c**–**g** Quantitative RT-PCR analysis of *RUNX2*, *Sp7*, *Alp*, *Col1a*, *FXR* expression in BMSCs after 3 and 7 days of osteoblastic differentiation (*n* = 3). **h** Western blot analysis of FXR, RUNX2 and Sp7 during osteoblast differentiation in the mouse BMSCs. Samples were collected at 0, 1, 3, 7, and 10 days after differentiation. **i** Western blot analysis of FXR in femoral osteoblasts at mice different developmental stages. **j, k** Representative immunofluorescence images (**j**) and quantification (**k**) of FXR (red) positive cells and Osteoblast marker (Sp7, green) on embryonic day 18.5, 1 week, 2 weeks and 4 weeks femur from C57BL/6 J mice. The nuclei were visualized via DAPI (blue) staining. GP means Growth Plate. Scale bar = 100 μm. *n* = 5. *P < 0.05, **P < 0.01, ***P < 0.001. Data are represented as mean ± SEM

To investigate the potential roles of NRs in osteoblast differentiation, we conducted RNA- sequencing analysis on total RNA extracted from BMSCs following osteoblastic differentiation (Fig. 1a). As depicted in Fig. 1b, this analysis revealed significant upregulation of multiple osteogenesis-related genes, such as *RUNX2, Sp7, Spp1, and Ibsp*. Furthermore, except the undetected NRs, the farnesoid X receptor was specifically upregulated during BMSCs osteoblastic differentiation (Fig. 1b). Based on these findings, we hypothesized that FXR might play a role in osteogenic differentiation. To validate this hypothesis, we induced osteoblast differentiation of BMSCs in vitro and observed a time-dependent increase in the expression of osteoblast marker genes such as *RUNX2*, *Osterix (*Osx, also known as Sp7*)*, alkaline phosphatase (*ALP*), and collagen type 1 alpha 1 chain (*Col1a*) (Fig. 1c–f). Additionally, we found that the expression of FXR exhibited a similar trend (Fig. 1g, h). Given the association between bone formation and osteoblast differentiation, we further explored the role of FXR in bone development by analyzing the expression levels of osteoblast differentiation marker genes Sp7 and FXR at embryonic day 18.5, week 1, week 2, and week 4 stages. Western blot and Immunofluorescence staining analysis revealed time dependent increase of FXR expression in the femurs during bone development (Fig. 1i–k). Taken together, these results demonstrated that FXR might have a vital physiological role in osteogenic differentiation and bone formation.

### Deletion of FXR in MSCs causes delayed bone development and osteoporosis

**Fig. 2:**
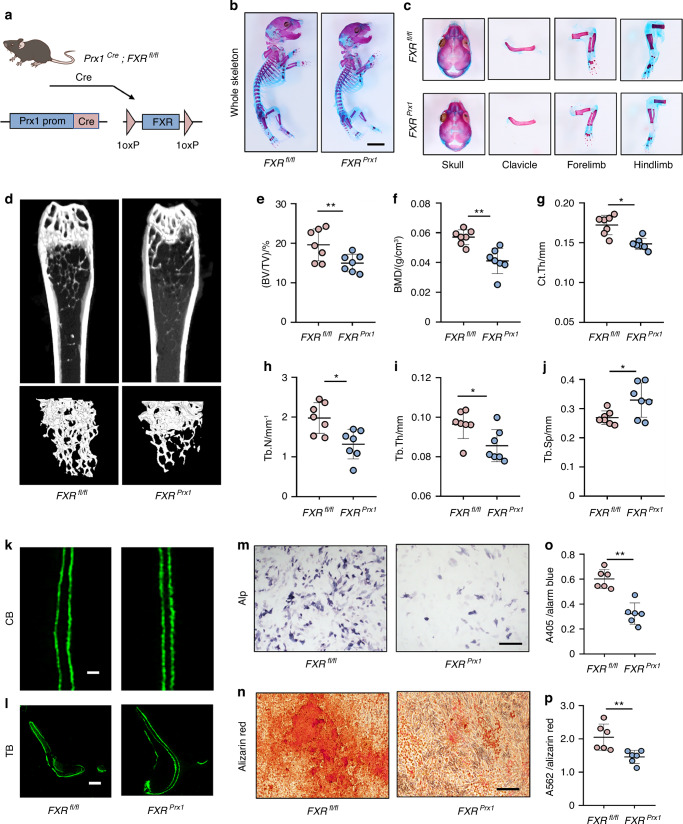
Deletion of FXR in MSCs causes delayed bone development and osteoporosis. **a** Illustration of osteoblast lineage-specific FXR knockout mice, which were generated via the crossbreeding of *FXR*^*fl/fl*^ mice with *Prx1-Cre* transgenic mice. **b**, **c** Representative double-stained with alcian blue and alizarin red S image of skeletal preparations from *FXR*^*fl/fl*^ and *FXR*^*Prx1*^ newborns. Scale bar = 5 mm. **d**–**j** Representative micro-CT images of distal femurs in 8-week-old *FXR*^*fl/fl*^ and *FXR*^*Prx1*^ mice with morphometric analysis of bone volume per tissue volume (BV/TV), bone mass density (BMD), trabecular thickness (Tb.Th), trabecular number (Tb.N), trabecular spacing (Tb.Sp) and cortical thickness (Ct.Th) (*n* = 7). **k**–**l** Representative images of calcein double labeling from *FXR*^*fl/fl*^ and *FXR*^*Prx1*^ mice (*n* = 4). Scale bar = 20 μm. CB means cortical bone. TB means trabecular bone. **m**–**p** Representative images and quantitative analysis of Alp staining and Alizarin red staining of BMSCs from *FXR*^*fl/fl*^ and *FXR*^*Prx1*^ mice. Scale bar = 50 μm (*n* = 6). *P < 0.05, **P < 0.01. Data are represented as mean ± SEM

To further investigate the physiological role of FXR in the determination of mesenchymal stem cell (MSC) commitment to the osteoblast lineage, we generated *Prx1-cre; FXR*^*fl/fl*^ mice (hereafter referred to as *FXR*^*prx1*^ *mice*) by crossing *FXR*^*fl/fl*^ *mice* with *Prx1-Cre* (*Prx1*^*Cre*^) mice (Fig. 2a). Prx1-Cre activity is primarily restricted to limbs and craniofacial mesenchyme cells (MSCs).^3^ Western blot and immunofluorescence staining analysis confirmed that FXR was successfully knocked out in BMSCs (Fig. S1a, b).

Newborn *FXR*^*prx1*^ mice exhibited a significantly smaller body size than their control littermates (Fig. S1c). Moreover, a delay in bone formation was observed in the skull, clavicles and limbs of newborn *FXR*^*prx1*^ mice (Fig. 2b, c). In addition, analysis of physical size revealed that 8-week-old *FXR*^*prx1*^ mice were dwarfed relative to their control littermates (Fig. S1d). To further elucidate the function of FXR within the skeletal system, micro-computed tomography (micro-CT) was employed to analyze the femurs of 8-week-old *FXR*^*prx1*^ mice and their control littermates. The analysis revealed that *FXR*^*prx1*^ mice displayed decreased BV/TV, BMD, and Ct.Th compared to age-matched control littermates (Fig. 2d–g). Subsequent analysis demonstrated that the reduction in Tb.N was accompanied by a decrease in Tb.Th and an increase in Tb.Sp in *FXR*^*prx1*^ mice, with this effect being independent of sex (Fig. 2h–j). To unravel whether the low bone mass in *FXR*^*prx1*^ mice was due to decreased bone formation. Histomorphometric analysis revealed that the osteoblast number/bone surface (N.Ob/BS) was significantly decreased in *FXR*^*prx1*^ mice (Fig. S1e, f). In addition, we observed a decrease in mineralization apposition rate (MAR) and bone formation rate (BFR) in *FXR*^*prx1*^ mice compared to their control littermates, as determined by fluorescent labeling of the mineralizing front (Fig. 2k, l and Fig. S1i, j). Additionally, FXR knockout did not affect the proliferation and differentiation of chondrocytes (Fig. S2a–e). Similarly, there was no significant difference in osteoclastogenesis of *FXR*^*prx1*^ mice compared to age-matched control littermates (Fig. S1g, h). These findings collectively demonstrate that specifically deficiency of FXR in MSCs leads to low bone mass and a delay in bone development.

As is well-established, osteoblasts originate from BMSCs. Consequently, we sought to investigate whether FXR deficiency influences the osteogenic potential of BMSCs. Primary BMSCs were isolated from the bone marrow of *FXR*^*prx1*^ mice and their control littermates and subsequently cultured in an osteogenic medium. We observed that both osteoblast differentiation (measured by ALP staining and activity) and calcification (measured by ARS staining) were significantly impaired in the *FXR*^*prx1*^ mice (Fig. 2m–p). However, FXR knockout did not affect the proliferation of BMSCs (Fig. S1k). Our findings suggest that FXR regulates bone mass by promoting osteoblast differentiation.

### Deletion of FXR in osteoclasts did not affect delayed bone development and osteoporosis phenotype

**Fig. 3:**
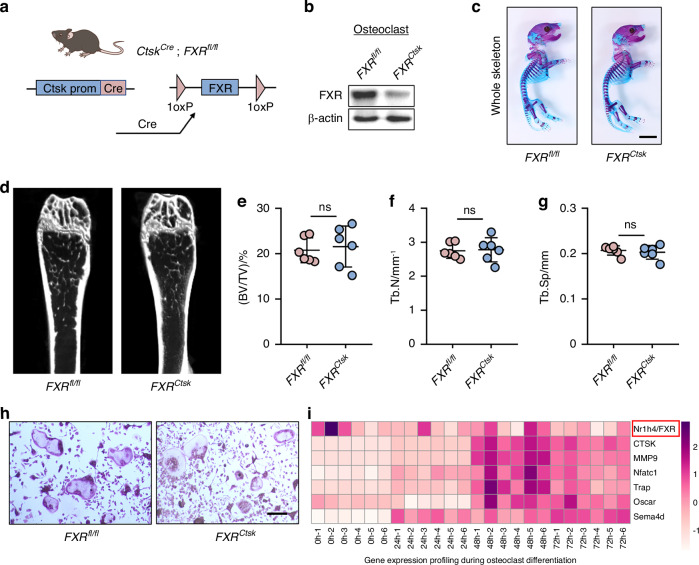
Deletion of FXR in osteoclasts did not affect delayed bone development and osteoporosis phenotype. **a** Illustration of osteoclast lineage-specific FXR knockout mice, which were generated via the crossbreeding of *FXR*^*fl/fl*^ mice with *Ctsk-Cre* transgenic mice. **b** Western blot analysis of FXR in BMMs from 4-week-old male *FXR*^*fl/fl*^ and *FXR*^*ctsk*^ mice cultured with M-CSF and RANKL for 7 days. **c** Representative double-stained with alcian blue and alizarin red S image of skeletal preparations from *FXR*^*fl/fl*^ and *FXR*^*Ctsk*^ mice newborns. Scale bar = 5 mm. **d**–**g** Representative micro-CT images of distal femurs in 8-week-old *FXR*^*fl/fl*^ and *FXR*^*Ctsk*^ mice with morphometric analysis of bone volume per tissue volume (BV/TV), trabecular number (Tb.N), trabecular spacing (Tb.Sp). *n* = 6. **h** Representative TRAP staining images of BMM from *FXR*^*fl/fl*^ and *FXR*^*Ctsk*^ mice. Scale bar = 50 μm. **i** Heatmap of FXR and osteoclast-related genes mRNA expression in osteoclastic differentiation (*n* = 6). ns no significant difference. Data are represented as mean ± SEM

Throughout life, the development and remodeling of bone depend on delicate equilibrium between bone formation by osteoblasts and bone resorption by osteoclasts. Deviations from this balance, such as reduced bone formation, increased bone resorption, or a combination of both can lead to bone deformity and the loss of bone mass. To investigate the potential role of FXR in this process, we generated osteoclast-specific FXR knockout mice (hereafter referred to as *FXR*^*Ctsk*^ *mice*) by crossing *FXR*^*fl/fl*^ *mice* with cathepsinK-Cre (*Ctsk-Cre*) mice, a line in which Cre activity is primarily restricted to osteoclasts^26,27^ (Fig. 3a). Western blot analysis and immunofluorescence staining confirmed the successful knockout of FXR in osteoclast (Fig. 3b and Fig. S3a). Alcian blue and Alizarin red S staining revealed no developmental bone deformities in *FXR*^*Ctsk*^ newborn mice compared to their control littermates (Fig. 3c and Fig. S3b). Furthermore, physical size analysis demonstrated that 8-week-old *FXR*^*Ctsk*^ *mice* did not exhibit dwarfism relative to their control littermates (Fig. S3c). In addition, we found that 8-week-old *FXR*^*Ctsk*^ *mice* showed no differences in BV/TV, Tb.N, and Tb.Sp compared to age-matched control littermates (Fig. 3d–g). In vitro, FXR-knockout did not affect the formation of multinucleate osteoclasts (Fig. 3h). Consistent with this, publicly available data from the Gene Expression Omnibusthe Database (GSE176265) indicated that the expression level of FXR did not change significantly during osteoclast differentiation (Fig. 3i). Collectively, our results suggest that deficiency of FXR in osteoclasts may not be a primary cause of skeletal deformities.

### Osteoblasts-specific deficiency of FXR reduced osteogenesis

To further elucidate whether the abnormal osteogenesis observed in *FXR*^*prx1*^ mice resulted from a primary defect in osteoblast development, we generated osteoblast-specific FXR-knockout mice (hereafter referred to as *FXR*^*OCN*^ mice) by crossing *FXR*^*fl/fl*^ mice with *OCN-Cre* mice (Fig. S4a). OCN-Cre expression is known to be primarily restricted to osteoblasts.^28^ Western blot and immunofluorescence staining analysis confirmed the successful knockout of FXR in osteoblasts (Fig. S4b, c). Analysis of physical size revealed that 8-week-old *FXR*^*OCN*^ mice exhibited dwarfism relative to their control littermates (Fig. S4d). To further investigate the effects of osteoblast-specific FXR-deficiency on the skeletal system, we employed micro-CT to analyze the skeletal elements within the femurs of *FXR*^*OCN*^ mice and their control littermates. We observed that 8-week-old *FXR*^*OCN*^ mice displayed decreased BV/TV, BMD, and Ct.Th compared to age-matched control littermates (Fig. S4e–h). Subsequent analysis demonstrated that *FXR*^*OCN*^ mice exhibited a decrease in Tb.Th and Tb.N but an increase in Tb.Sp (Fig. S4i–k). Since osteogenesis is associated with osteoblast differentiation and calcification, we cultured calvarial cells from *FXR*^*OCN*^ mice and *FXR*^*fl/fl*^ mice in an osteogenic medium to assess the role of FXR in these processes. We observed that both osteoblast differentiation and calcification were significantly impaired in the *FXR*^*OCN*^ group (Fig. S4l–o). Collectively, these findings indicate that FXR is essential for the differentiation and function of osteoblasts.

### FXR stabilizes RUNX2 by inhibiting ubiquitination

We next sought to address the mechanism how FXR controls bone mass and osteoblast differentiation. Osteoblast differentiation is achieved by activating a transcriptional network in which Runx2, and Sp7 have vital roles.^28^ *FXR*^*prx1*^ mice exhibited osteopenia and specific phenotypes characteristic of partial RUNX2 loss-of-function (Fig. 2b, c). Moreover, previous studies have demonstrated that activation of FXR increased RUNX2 activity.^10^ Therefore, we hypothesized that FXR might influence bone development and osteogenic differentiation by regulating RUNX2.

**Fig. 4:**
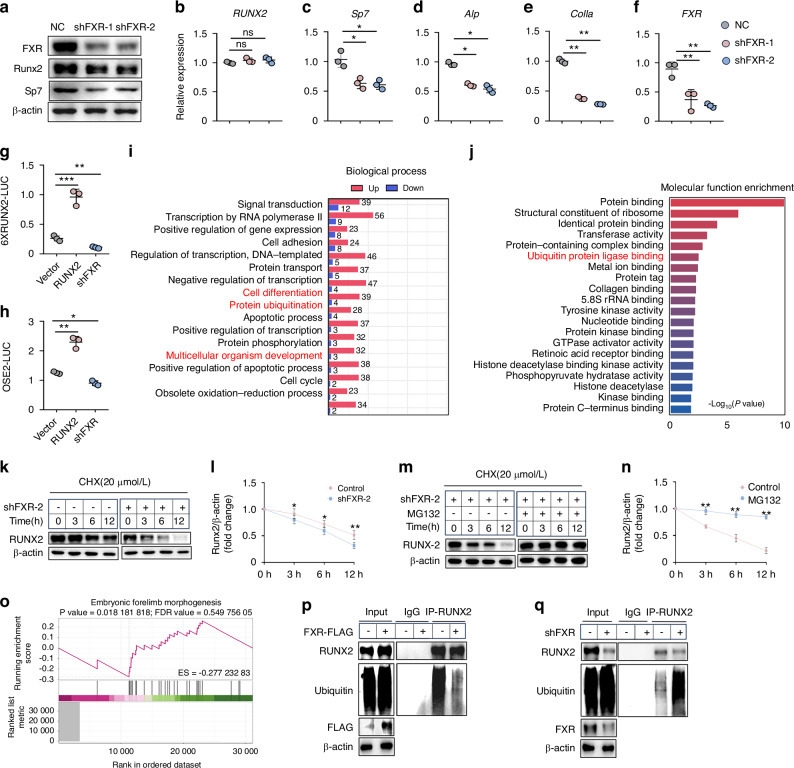
FXR stabilizes RUNX2 by inhibiting ubiquitination. **a** Western blot analyses of FXR, RUNX2, Sp7 in BMSCs after 4 days of osteoblastic differentiation transfection with or without FXR shRNA. **b-f** Quantitative RT-PCR analysis of RUNX2, Sp7, Alp, Col1a, FXR expression in BMSCs after 4 days of osteoblastic differentiation transfection with or without FXR shRNA (*n* = 3). **g**, **h** 6XRUNX2 and OSE2-luciferase activity was determined in C3H10T1/2 cells cotransfected with RUNX2 cDNA or FXR shRNA (*n* = 3). **i**, **j** Gene Ontology (GO) enrichment analysis of differentially expressed genes in (**i**) Biological Process and (**j**) Molecular Function. **k** Western blot analyses of RUNX2 expression in C3H10T1/2 cells, which were transfected with or without FXR shRNA for 48 h, and were treated with cycloheximide (CHX) for the indicated time. **l** Quantitative analysis of RUNX2 protein level (*n* = 3). **m**, **n** Western blot analyses of RUNX2 expression in C3H10T1/2 cells, which were treated with cycloheximide (CHX) for the indicated time in the transfection of FXR shRNA, and were treated with MG132 (10 μmol/L) for 6 h. **n** Quantitative analysis of RUNX2 protein level (*n* = 3). **o** The embryonic forelimb morphogenesis pathway related genes were obtained via Gene Set Enrichment Analysis (GSEA). **p** After C3H10T1/2 cells were transfected with Vector or FXR-FLAG for 48 h, lysed, immunoprecipitated with anti-RUNX2 conjugated protein A/G magnetic beads, and immunoblotted with anti-ubiquitin antibody. **q** After C3H10T1/2 cells were transfected with control shRNA or FXR shRNA for 48 h, lysed, immunoprecipitated with anti-RUNX2 conjugated protein A/G magnetic beads, and immunoblotted with anti-ubiquitin antibody. *P < 0.05, **P < 0.01, ***P < 0.001, ns no significant difference. Data are represented as mean ± SEM

To investigate this hypothesis, BMSCs were subjected to FXR activity blockade using short hairpin RNA (shRNA) lentiviral infection and subsequently cultured in osteogenic medium. We initially analyzed the expression of several osteogenesis-related genes, including *RUNX2*, *Sp7*, *ALP* and *Col1a* in BMSCs. As depicted in Fig. 4a, the protein levels of RUNX2 and Sp7 were markedly reduced following FXR knockdown. Immunofluorescence staining analysis indicated that FXR knockout reduced RUNX2 expression in osteoblast (Fig. S5m). Consistent with these findings, the mRNA levels of *Sp7*, *ALP*, and *Col1a* were significantly decreased (Fig. 4c–f). In addition, we conducted dual-luciferase reporter assays using OSE2-luciferase or 6XRUNX2-luciferase and Renilla in C3H10T1/2 cells. The results indicated that FXR knockdown significantly inhibited the activation of OSE2-luciferase and 6XRUNX2-luciferase (Fig. 4g, h). To further explore the potential impact of pharmacological FXR inactivation on osteogenic differentiation, we employed (Z)-Guggulsterone, an FXR inhibitor. Similarly, (Z)-Guggulsterone markedly inhibited the expression of osteogenesis-related genes (Fig. S5a, c–e). In contrast, overexpression of FXR significantly enhanced the expression of these genes (Fig. S5f, h–j). However, alterations in FXR expression did not affect the transcriptional level of RUNX2 (Fig. 4b, Fig. S5b, g). Therefore, we hypothesized that FXR might play a role in increasing the protein stability of RUNX2.

To analyze the effects of FXR deficiency on the expression profile of genes within osteoblasts, RNA sequencing was performed on total RNA extracted from BMSCs isolated from *FXR*^*prx1*^ mice and their *FXR*^*fl/fl*^ littermates. Both groups of cells were cultured under osteogenic medium conditions (Fig. S6a). This analysis revealed that 69 genes were downregulated by more than 1.5-fold, while 615 genes were upregulated by more than 1.5-fold in the *FXR*^*prx1*^ mice compared to the *FXR*^*fl/fl*^ littermates (Fig. S6b). Gene Ontology (GO) analysis applied to the differentially expressed genes indicated that these genes were significantly enriched for functional categories associated with multicellular organism development, cell differentiation, and embryonic forelimb morphogenesis (Fig. 4i, o). These findings are consistent with the skeletal defect observed in *FXR*^*prx1*^ mice, such as shortened limbs and decreased bone density. Interestingly, GO analysis also identified an enrichment of genes associated with protein ubiquitination and ubiquitin protein ligase binding (Fig. 4i, j and Fig. S6c). This finding prompted us to investigate the potential impact of FXR on the protein stability of RUNX2 using a protein synthesis inhibitor chase assay with cycloheximide (CHX). The results demonstrated that the half-life of RUNX2 protein was significantly reduced in C3H10T1/2 cells with FXR knockdown (Fig. 4k, l), suggesting that FXR may regulate RUNX2 through a post-translational mechanism. The ubiquitin-proteasome system is a well-established cellular pathway responsible for the targeted degradation of most intracellular proteins, thereby regulating a diverse range of cellular activities.^29^ To further determine whether the decreased stabilization of RUNX2 observed with FXR- knockdown was mediated through the ubiquitination pathway, C3H10T1/2 cells were treated with MG132, a proteasome inhibitor. This treatment resulted in a substantial increase in the protein level of RUNX2 within these cells (Fig. 4m, n), suggesting that ubiquitin-dependent degradation plays a role in maintaining RUNX2 homeostasis. To elucidate the potential role of FXR in mediating the ubiquitination of RUNX2, an immunoprecipitation (IP) assay was performed to detect the ubiquitination levels of RUNX2 in C3H10T1/2 cells with FXR-overexpression. This experiment revealed that overexpression of FXR significantly reduced the ubiquitination of RUNX2 (Fig. 4p). Conversely, FXR-knockdown markedly increased the ubiquitination of RUNX2 (Fig. 4q). Similarly, knocking out FXR in BMSCs and osteoblast increased the ubiquitination of RUNX2 (Fig. S5k, l). Taken together, these data suggest that FXR may promote the stabilization of RUNX2 by reducing its ubiquitination.

### Thoc6 inhibits osteoblast differentiation by interacting with RUNX2

**Fig. 5:**
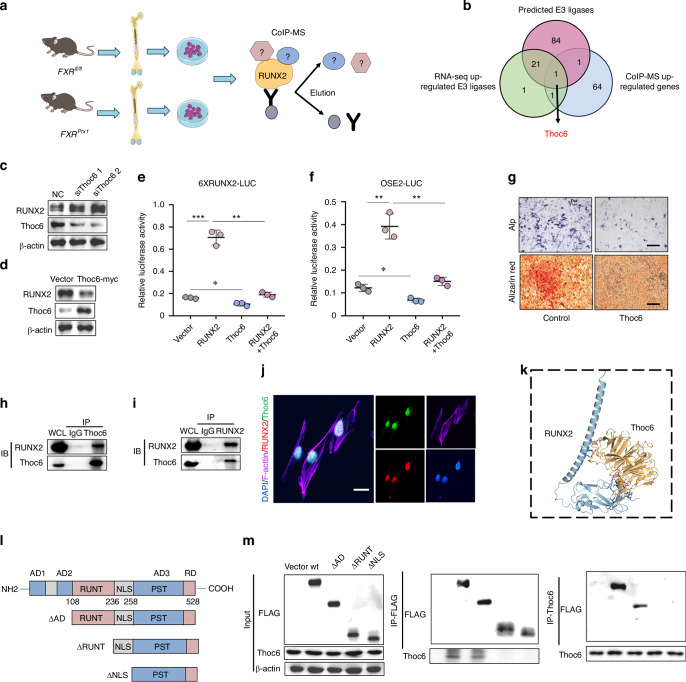
Thoc6 inhibits osteoblast differentiation by interacting with RUNX2. **a** Schematic graph of the strategy to identify RUNX2 interacting protein which were regulated by FXR. **b** Venn diagram representing the intersection gene between Predicted E3 ligases, RNA-seq up-regulated E3 ligases and IP-MS up-regulated proteins. **c** Western blot analyses of RUNX2 expression in C3H10T1/2 cells, which were transfected with control siRNA or Thoc6 siRNA for 48 h. **d** Western blot analyses of RUNX2 expression in C3H10T1/2 cells which were transfected with Vector or Thoc6 cDNA for 48 h. **e**, **f** 6XRUNX2 and OSE2-luciferase activity was determined in C3H10T1/2 cells transfected with RUNX2 or Thoc6 cDNA (*n* = 3). **g** Representative images of Alp staining and Alizarin red staining of BMSCs which were infected with Thoc6 lentivirus. Scale bar = 50 μm. **h**, **i** BMSCs were lysed after 4 days of osteoblastic differentiation, immunoprecipitated with anti-IgG control, anti-Thoc6 (**h**) or anti-RUNX2 (**i**) antibody conjugated protein A/G magnetic beads, and immunoblotted with the indicated antibodies. WCL means whole cell lysate. **j** Representative immunofluorescent staining of RUNX2 and Thoc6, which was used to evaluate the subcellular location in BMSCs. Scale bar = 5 μm. **k** Optimized binding modes of RUNX2 and Thoc6 with the lowest binding energy generated by the HDOCK protein-docking algorithm**. l** Schematic diagram of the domain structure of Runx2 and its N-terminal deletion mutants. **m** Interaction between THOC6 and the N-terminal amino acid deletion of RUNX2 in C3H10T1/2 cells. Whole cell extracts from C3H10T1/2 cells transfected with expression vectors for Thoc6 and WT FLAG-Runx2 or various FLAG-Runx2 N-terminal deletion mutants (FLAG-ΔAD, FLAG-ΔRUNT, and FLAG-ΔNLS) were immunoprecipitated FLAG or Thoc6 antibody, followed by Western blot using Thoc6 or FLAG antibody. *P < 0.05, **P < 0.01, ***P < 0.001. Data are represented as mean ± SEM

To identify potential E3 ubiquitin ligases that cooperate with FXR to regulate the ubiquitination of RUNX2, we employed a bioinformatic approach. The RUNX2 protein sequence was queried as a potential substrate within the UbiBrowser database (http://ubibrowser.ncpsb.org.cn/ubibrowser_v3/).^30,31^ This analysis revealed the identification of 6 E3 ligases and the prediction of 107 additional E3 ligases that might interact with RUNX2 (Fig. S7a). Notably, 24 of these predicted E3 ligases were also found to be upregulated in the RNA sequencing data (Fig. S7b). We subsequently employed co-immunoprecipitation (Co-IP) coupled with mass spectrometry to identify proteins that potentially interact with RUNX2 in vitro (Fig. 5a and Fig. S7c). This analysis identified 67 proteins exhibiting increased binding to RUNX2 following FXR knockout, potentially serving as RUNX2-binding partners. Interestingly, Thoc6 was identified within all three sets of analyzed data (Fig. 5b). Furthermore, the knockdown of Thoc6 resulted in a significant increase in the protein level of RUNX2 (Fig. 5c). Conversely, overexpression of Thoc6 led to a marked reduction in the protein expression of RUNX2 (Fig. 5d) and a diminished activation of both OSE2-luciferase and 6XRUNX2-luciferase reporters (Fig. 5e, f). Consistent with these observations, the mRNA expression levels of Sp7, Alp, and Col1a were significantly decreased upon overexpression of Thoc6 (Fig. S7d–g). Moreover, Thoc6 overexpression significantly impeded the processes of osteogenic differentiation and mineralization (Fig. 5g and Fig. S7h, i). To investigate the mechanism by which Thoc6 regulates RUNX2, Co-IP assays demonstrated a physical interaction between Thoc6 and RUNX2 in C3H10T1/2 cells (Fig. 5h, i). Additionally, we observed a strong colocalization of Thoc6 with RUNX2 within the cell nucleus (Fig. 5j). To identify the specific binding sites within Thoc6 and RUNX2 involved in this interaction, a three-dimensional (3D) structural model of the Thoc6–RUNX2 complex was generated using the HDOCK protein-docking algorithm. The resulting model suggested that the interaction between RUNX2 and Thoc6 was mediated by the Runt domain of RUNX2 and by the WD repeat of Thoc6 (Fig. 5k and Fig. S8a). To further delineate the specific Thoc6-binding domain within the RUNX2 molecule, C3H10T1/2 cells were co-transfected with plasmids expressing Thoc6 and either wild-type (WT) FLAG-tagged RUNX2 or various FLAG-tagged RUNX2 N-terminal deletion mutants (Fig. 5l). As shown in Fig. 5m, deletion of the N-terminal 108 amino acids of RUNX2 (ΔAD) did not affect its ability to bind to Thoc6. However, further deletions encompassing amino acid residues 236 (ΔRUNT) and 258 (ΔNLS) completely abolished the interaction between RUNX2 and Thoc6. These results demonstrated that Thoc6 inhibits osteoblast differentiation by interacting with RUNX2 and the N-terminal region amino acids 108–236 (RUNT domain) is essential for RUNX2 to bind to Thoc6.

### Activation of FXR decreases Thoc6 expression

**Fig. 6:**
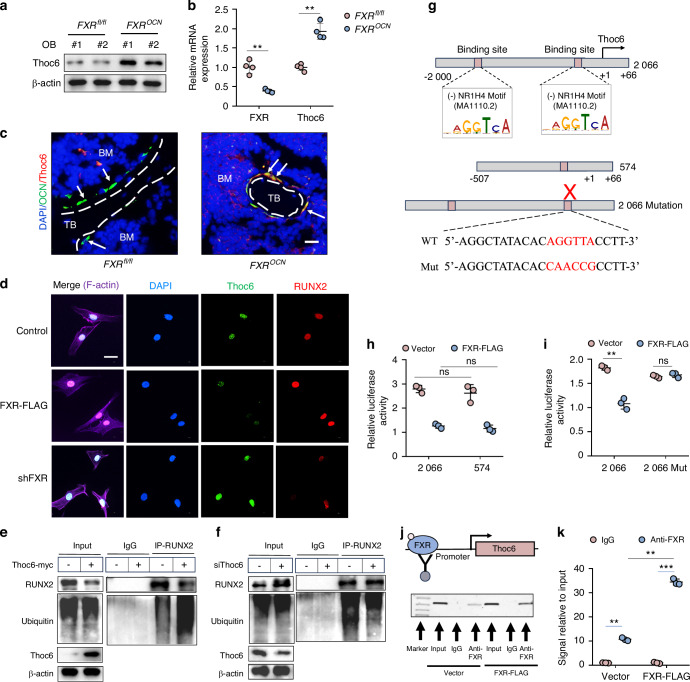
Activation of FXR suppresses Thoc6 expression. **a**, **b** Western blot and Quantitative RT-PCR analysis of Thoc6 expression in osteoblast cells from *FXR*^*fl/fl*^ and *FXR*^*OCN*^ mice after 4 days of osteoblastic differentiation (*n* = 4). **c** Immunofluorescence assay for Thoc6 expression in osteoblast (OCN-a marker of osteoblasts) of *FXR*^*fl/fl*^ and *FXR*^*OCN*^ mice (*n* = 3). Scale bars, 50 μm. BM means bone marrow. TB means trabecular bone. **d** Immunofluorescence assay for expression of RUNX2 and Thoc6 in osteoblast transfection with FXR cDNA or FXR shRNA. Scale bar = 10 μm. **e** After C3H10T1/2 cells were transfected with Vector orThoc6-myc for 48 h, lysed, immunoprecipitated with anti-RUNX2 conjugated protein A/G magnetic beads, and immunoblotted with anti-ubiquitin antibody. **f** After C3H10T1/2 cells were transfected with control siRNA or Thoc6 siRNA for 48 h, lysed, immunoprecipitated with anti-RUNX2 conjugated protein A/G magnetic beads, and immunoblotted with anti-ubiquitin antibody. **g** A schematic representation of the Thoc6 promoter region showing potential FXR (NR1H4) binding sites. **h**, **i** C3H10T1/2 cells were co-transfected with one of these designated constructs (constructs 2 066, construct 574, 2 066 Mut with mutated one FXR binding sites) and with FXR cDNA construct for 48 h, and then luciferase activity was measured and normalized (*n* = 3). **j** Schematic diagram depicting ChIP assays. **k** C3H10T1/2 cells were transfected with FLAG-tagged FXR cDNA for 72 h. ChIP assays were performed using anti-FLAG antibody. The Standard PCR products were run and scanned (left panel). The histogram was presented as quantification of the PCR results (right panel) (*n* = 3). **P < 0.01, ***P < 0.001, ns no significant difference. Data are represented as mean ± SEM

To further explore the regulatory mechanisms underlying FXR’s influence on Thoc6 expression, we measured the expression levels of Thoc6 at both the mRNA and protein levels. Western blot analyses (Fig. 6a), quantitative RT-PCR (Fig. 6b) and immunofluorescence staining (Fig. 6c) revealed that FXR knockout significantly increased Thoc6 expression. Similarly, immunofluorescence staining demonstrated that FXR overexpression inhibited the expression of Thoc6 while enhancing the nuclear accumulation of RUNX2 and vice versa (Fig. 6d). To investigate the potential role of Thoc6 in regulating RUNX2 ubiquitination, Co-IP assays were performed to detect the ubiquitination levels of RUNX2 in C3H10T1/2 cells. These experiments revealed that overexpression of Thoc6 significantly increased the ubiquitination levels of RUNX2 (Fig. 6e) and vice versa (Fig. 6f). Consistent with these results, overexpression of Thoc6 could reversed FXR reducing the ubiquitination of RUNX2 (Fig. S8f) and vice versa (Fig. S8g). Furthermore, we analyzed the effect of FXR on Thoc6 promoter activity using a dual-luciferase reporter assay. Notably, the promoter activity of Thoc6 was significantly reduced by FXR overexpression, suggesting that FXR transcriptionally regulates Thoc6 expression (Fig. 6h). Bioinformatic analysis of the Thoc6 promoter sequence identified two putative FXR binding sites upstream of the transcriptional start site (Fig. 6g). To investigate whether FXR directly binds to these predicted binding sites, we constructed short Thoc6 promoter constructs fused to a luciferase reporter gene (Fig. 6g). Constructs containing either both predicted Thoc6 binding sites (2 066) or only one site (574) relative to the transcriptional start site still exhibited a significant reduction in promoter activity upon FXR overexpression (Fig. 6h), but there was no discernible difference between constructs 2 066 and 574. These results suggested a crucial role for the second predicted FXR binding site. In line with these findings, a construct with point mutations within the second FXR binding site prevented the FXR-induced reduction of Thoc6 promoter activity (Fig. 6i). Moreover, ChIP assays were conducted to further confirm the binding of FXR to the Thoc6 promoter. These experiments revealed that a fragment containing the second predicted FXR binding site was specifically immunoprecipitated by anti-FLAG antibodies (Fig. 6j, k), indicating that FXR directly mediates the repression of Thoc6 promoter activity. Collectively, these results demonstrate that activation of FXR leads to a decrease in Thoc6 expression.

### Pharmacological activation of FXR promoted osteoblast differentiation and partially prevented bone mass loss induced by OVX

**Fig. 7:**
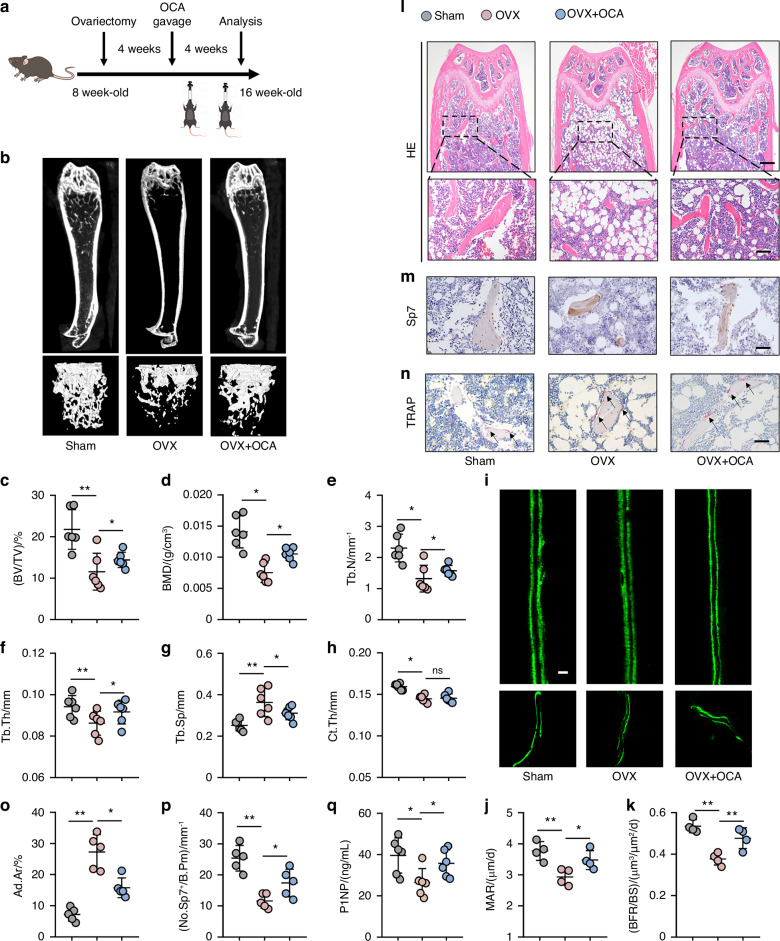
Pharmacological activation of FXR promoted osteoblast differentiation and partially prevented bone mass loss induced by OVX. **a** Schematic representation of experimental design of OCA treatment in OVX mice. **b**–**h** Representative micro-CT images of distal femurs in Sham, OVX, OVX treated with OCA groups mice with morphometric analysis of bone volume per tissue volume (BV/TV), bone mass density (BMD), trabecular thickness (Tb.Th), trabecular number (Tb.N), trabecular spacing (Tb.Sp) and cortical thickness (Ct.Th) (*n* = 6). **i**–**k** Representative images of calcein double labeling and quantitative analysis of mineralization apposition rate (MAR) and bone formation rate (BFR) from Sham, OVX, OVX treated with OCA groups mice (*n* = 4). Scale bar = 20 μm. CB means cortical bone. TB means trabecular bone. **l**, **o** Representative images of hematoxylin and eosin (HE) staining (**l**) in the femurs area from Sham, OVX, OVX treated with OCA groups. Adipocyte area (**o**) was analyzed in accordance with HE staining (*n* = 5). Scale bar = 500 μm. **m**, **p** Immunohistochemistry staining (**m**) and quantification (**p**) of Sp7 (Osx, an osteoblastic lineage cell marker) in the femurs of mice (*n* = 5). Scale bar = 50 μm. **q** Elisa analysis of serum PINP (ng/ml) from Sham, OVX, OVX treated with OCA groups mice (*n* = 6). **n** TRAP staining of femur area from sham, OVX, OVX treated with OCA groups (*n* = 6). Scale bar = 50 μm. *P < 0.05, **P < 0.01. Data are represented as mean ± SEM

FXR has emerged as a potential therapeutic target for a variety of diseases, including primary biliary cholangitis (PBC)^32,33^, non-alcoholic fatty liver disease (NAFLD)^34^, and acute kidney injury.^35^ Therefore, we investigated the potential therapeutic effects of FXR pharmacological activation on osteoporosis. Obeticholic acid (OCA) is an orally bioavailable FXR agonist.^36^ Initially, we treated BMSCs isolated from wild-type mice with OCA at concentrations of 1 μmol/L and 5 μmol/L to induce osteogenic differentiation. We observed a significant induction in the mRNA expression levels of osteogenic genes such as *Sp7*, *Alp*, and *Col1a1* in OCA-treated groups (Fig. S8i–l). Additionally, OCA treatment resulted in a robust dose-dependent increase in the protein expression of RUNX2 (Fig. S8h). Based on the in vitro findings, we established an osteoporosis mouse model by OVX and subsequently treated the OVX mice with OCA (5 mg/kg/day) for one month (Fig. 7a). Notably, OCA treatment significantly restored the decreased bone mass observed in the femurs of OVX mice. Micro-CT analysis revealed increased BV/TV, BMD, Tb.N, and Tb.Th in the femurs of OCA-treated mice, while Tb.Sp was decreased (Fig. 7b–h). Fluorescent labeling of the mineralizing front analysis indicated increased MAR and BFR of OCA-treated mice (Fig. 7i–k). Histomorphometry analysis further demonstrated that OCA treatment alleviated the OVX-induced accumulation of adipocytes in the bone marrow cavity (Fig. 7l, o). Furthermore, our data demonstrated a significant increase in the number of osteoblastic lineage cells (Fig. 7m, p) and the bone formation biomarker N-terminal propeptide of type I procollagen (PINP) in OCA-treated mice compared to the OVX group (Fig. 7q). Notably, the number of osteoclasts in OCA-treated mice was not significantly different from the OVX group (Fig. 7n and Fig. S8m). Collectively, these findings suggest that pharmacological activation of FXR promotes osteoblast differentiation both in vitro and in vivo. This data supports the potential of FXR as a promising therapeutic target for the treatment of osteoporosis.

## Discussion

Previous studies have demonstrated that members of the nuclear receptor superfamily, such as the estrogen receptor (ER)^37^, vitamin D receptor (VDR)^38,39^, peroxisome proliferator-activated receptor (PPARγ)^40^ and Liver X receptors (LXR)^41^, play vital roles in regulating bone homeostasis. While the function of FXR in bone homeostasis has not been extensively studied, our findings provide genetic evidence that inactivation of FXR within the osteoblast lineage results in impaired bone formation and reduced bone mass. Mechanistically, we elucidated that FXR could suppress Thoc6-mediated ubiquitination of RUNX2 by inhibiting the expression of Thoc6. This stabilization of RUNX2 counteracted ubiquitin-dependent proteasomal degradation. Furthermore, pharmacological activation of FXR could alleviate osteoporosis in ovariectomized mice. Collectively, these data underscore the crucial role of FXR in osteogenesis.

FXR, a member of the nuclear receptor superfamily activated by endogenous bile acid ligands, plays a role in various cellular processes. However, the specific functions of FXR within the skeletal system remain largely unknown. While a previous study suggested that FXR depletion enhances osteoclast differentiation^9^, we found that osteoclast differentiation and bone mass are normal in *FXR*^*Ctsk*^ mice (Fig. 3d–h). Cho et al. demonstrated that FXR deletion in macrophages significantly increased RANKL-dependent NFATc1 induction only within 24 h, suggesting a potential role for FXR in regulating the early stages of osteoclast differentiation from precursors.^9^ As differentiation and maturation are critical processes impacting osteoclast functionality^42^, the discrepancy with previous findings may be attributed to the relatively late activation of *Ctsk-Cre* in differentiated osteoclasts. Further investigation is required to elucidate the precise role of FXR in osteoclasts. Our future studies will focus on targeting FXR earlier in macrophage/monocyte precursors to reveal its potential role in osteoclast commitment.

The THO complex, a component of the TREX (transcription/export) complex, plays a crucial role in transcriptional elongation, nuclear RNA export, and genome stability.^43^ However, despite being a member of the THO complex,^44^ the molecular functions of Thoc6 remain relatively unexplored. Previous studies have demonstrated that homozygous missense mutations in Thoc6 lead to intellectual disability and skeletal anomalies.^45^ While no prior studies have reported the involvement of Thoc6 in mediating the ubiquitination of other proteins, our current research identified RUNX2 as a potential substrate for Thoc6. Thoc6 knockdown resulted in lower levels of RUNX2 ubiquitination (Fig. 6f). Structural analyses generated using AlphaFold revealed that THOC6 is a WD40-repeat protein (WDR) comprising seven WD40-repeat domains (Fig. S8b, c). WDRs are arranged in a distinctive β-propeller (β-Prp) fold, consisting of six to eight “blades” that create a rigid interaction scaffold, facilitating protein-protein interactions and the formation of multiprotein complexes.^46^ Interestingly, WD40 β-propellers, when functioning as ubiquitin-binding domains (UBDs), can regulate substrates by promoting their ubiquitination and subsequent degradation by the proteasome.^47^ The CUL4-DDB1 ubiquitin E3 ligase is known to regulate numerous biological processes, including cell cycle progression, replication, differentiation, and DNA damage response.^48,49^ Moreover, multiple WDRs serve as substrate-specific adaptors for this E3 ligase, interacting with CUL4-DDB1 complexes to promote substrate recognition and polyubiquitination.^50–52^ Based on these findings, it is reasonable to propose a model in which CUL4-DDB1 ubiquitin E3 ligases utilize Thoc6 as molecular adaptors for the recognition and ubiquitination of RUNX2. Consistent with this hypothesis, our results suggested that Thoc6 interacts with DDB1 and CUL4B (Fig. S8d, e). However, it should be borne in mind that other mechanisms may also contribute to the assembly of Thoc6 with CUL4-DDB1, and further investigation is required to elucidate the exact molecular mechanisms involved. While we have identified RUNX2 as a critical substrate for Thoc6 in this study, it is highly conceivable that Thoc6 may have additional substrates that play roles in regulating osteogenesis.

Endochondral ossification is an important factor affecting bone development. To explore whether FXR could affect bone development by regulating endochondral ossification, we established in vitro model of endochondral ossification. However, the expression of FXR and Thoc6 did not change significantly during endochondral ossification (Fig. S2f–h). These results indicated that the mechanism of FXR regulating Thoc6 in osteogenesis may not apply to endochondral ossification.

Osteoporosis is a systemic skeletal disease that primarily affects postmenopausal women and the elderly, characterized by a loss of bone mass and increased bone fragility.^4,53^ Currently, available medications for the treatment of osteoporosis are categorized as anabolic (such as parathyroid hormone and recombinant human bone morphogenetic protein-2 [rhBMP-2]) and anti-resorptive (such as bisphosphonates, calcitonin, raloxifene and denosumab).^54^ However, their long-term usage is often limited by associated side effects. Head-to-head comparisons have indicated that anabolic agents demonstrate greater anti-fracture efficacy and result in larger increases in bone density compared to anti-resorptive drugs.^55^ Teriparitide, a synthetic form of naturally occurring parathyroid hormone, is currently the only FDA-approved anabolic or bone-building agent in the United States. However, the FDA recommends that cumulative use during a patient’s lifetime should not exceed 2 years.^56^ Our findings demonstrate that pharmacological activation of FXR activity increased bone mass by inducing osteoblast differentiation, suggesting an anabolic action of FXR in skeletal tissue. Bisphosphonates are the most widely used anti-resorptive agents. Long-term, continuous use of oral bisphosphonates is typically alternated with drug holidays lasting 1–2 years to minimize the risk of atypical femoral fractures.^56^ Therefore, targeting FXR in combination with teriparitide or bisphosphonates may have unexpected benefits in preventing bone loss.

OCA is the first FXR ligand to advance to the clinical stage.^57^ Currently, OCA is being investigated for its potential therapeutic applications in NASH and PBC.^58^ Interestingly, accumulating experimental evidence suggests a pathophysiological link between NAFLD and osteoporosis.^59^ Furthermore, the prevalence of osteoporosis in PBC patients is strikingly high, reaching up to 30%. In fact, osteoporosis is at least four times more prevalent in PBC patients compared to age- or gender-matched controls.^60^ The positive role of FXR in osteogenesis prompted us to explore its potential therapeutic applications in osteoporosis. Our research demonstrated that oral OCA administration reduced bone mass loss in the femurs of postmenopausal female mice. The potential for a single medication to protect against multiple diseases holds significant clinical value. Given the therapeutic efficacy of OCA in both bone formation and PBC, its future use in preventing osteoporosis or PBC with osteoporosis may be considered.

With the increasing global incidence of fractures, the development of effective medications for fracture prevention and treatment has become a pressing public health concern. While extensive efforts have been devoted to this area, there remains an urgent need for novel strategies in the development of anabolic drugs. Our findings highlight the direct osteogenic effects of FXR, suggesting that further exploration of FXR-targeted therapies for fracture treatment may be warranted. Subchondral bone has emerged as a promising therapeutic target in osteoarthritis (OA).^61^ The microstructural impairment of subchondral bone caused by osteoporosis is a contributing factor to cartilage damage in early osteoarthritis.^62,63^ Therefore, investigating whether targeting FXR at an early stage of OA could improve subchondral bone quality warrants further exploration. The side effects of OCA, such as cutaneous pruritus and nausea, are significant factors limiting its clinical application.^34^ Therefore, developing local drug delivery systems composed of biomaterials could serve as a more effective carrier for testing the effects of osteoblast-specific FXR in the treatment of osteoporosis.

Our findings suggest that FXR may be a promising therapeutic target for osteoporosis. However, it is important to acknowledge certain limitations of our study. One of the limitations of our study is that the promotion effect of FXR in osteoblast differentiation was mainly investigated in cellular and murine models, but not in human samples. Therefore, the efficacy of FXR in protecting against human osteoporosis warrants further exploration in future clinical trials. Additionally, while we investigated the effects of OCA on postmenopausal osteoporosis, it would be meaningful to extended our study to other types of osteoporosis, such as disuse or senile osteoporosis. Furthermore, it is well-established that the commitment of BMSCs to the osteoblast and adipocyte lineages is competitive.^64^ In our study, we observed that activation of FXR significantly inhibited the accumulation of fat within the bone marrow cavity of ovariectomized female mice (Fig. 7c, l), which also indirectly demonstrated the osteogenic effect of FXR. However, investigating whether FXR possesses the ability to directly inhibit the differentiation of BMSCs into adipocytes will be a focus of our future research.

**Fig. 8:**
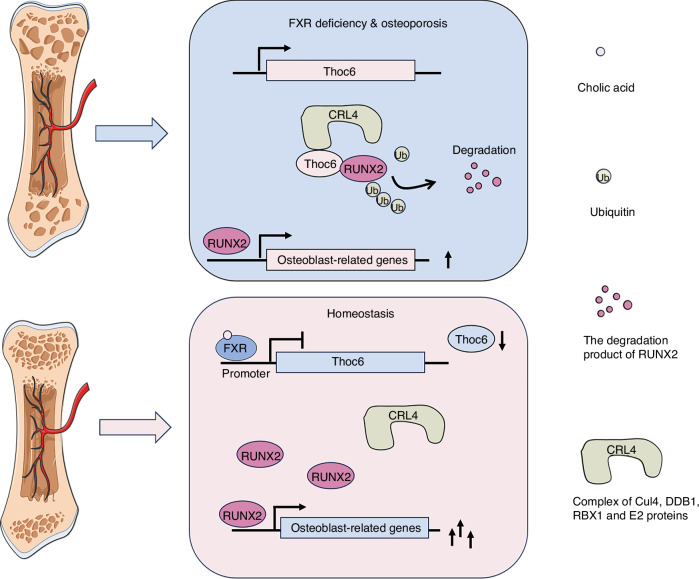
Graphic illustration of this study. Schematic illustration for the function of FXR in regulating osteoblast differentiation. The deficiency of FXR results in increased Thoc6 and RUNX2 is ubiquitinated excessively by Thoc6, followed by lysosomal degradation, consequently suppressing osteoblast differentiation leading to osteoporosis; During FXR sufficient, FXR promotes osteoblast differentiation by suppressing Thoc6 expression and reducing ubiquitination of RUNX2 to help maintain healthy bone homeostasis

In summary, our research demonstrates that FXR serves as a positive regulator of osteogenesis, influencing bone development and osteoblast differentiation. Mechanistically, FXR suppresses Thoc6-mediated ubiquitination of RUNX2 by inhibiting the expression of Thoc6, thereby stabilizing RUNX2 and countering ubiquitin-dependent proteasomal degradation. These findings collectively suggest that targeting FXR represents a promising therapeutic strategy for the treatment of osteoporosis (Fig. 8).

## Materials and methods

### Mice

All experiments were approved by the Animal Care and Use Committee of the Hebei Laboratory and Research Center for Food, Drug, and Medical Devices and were conducted in accordance with the National Institutes of Health Guidelines for the Care and Use of Laboratory Animals. FXR^*fl/fl*^ (B6.129 × 1(FVB)-Nr1h4tm1.2Gonz/J) and *Prx1-Cre* mice (Cre recombinase under the control of the Prx1 promoter) were obtained from the Jackson Laboratory (Bar Harbor, ME, USA). *Ctsk-Cre* mice (Cre recombinase under the control of the cathepsin-K promoter) were purchased from GemPharmatech (Nanjing, China). *OCN-Cre* mice (Cre recombinase under the control of the OCN promoter) were kindly provided by Dr. Ganggang Wang, Pudong Hospital, Fudan University. As shown in Fig. S9, genotypes were confirmed by PCR analysis of mouse tail DNA samples using the primer pairs listed in Table S1. Mice were maintained on a standard mouse diet and housed under a 12-h light/12-h dark cycle with controlled temperature (22 °C–24 °C) and humidity (50%–65%). All experiments were performed at similar times of the day to minimize any potential circadian rhythm effects on bone function.

### Antibodies, chemicals and reagents

The following antibodies were used: rabbit anti-RUNX2 (Abclonal, A11753), mouse anti-RUNX2 (Beyotime, AG1348), rabbit anti-Osterix (Abcam,ab209484), mouse anti-PRX1 (ORIGENE, TA803116S), mouse anti-FXR (R&D, PP-A9033A-00), rabbit anti-CTSK (Abcam, ab187647), rabbit anti-FXR (Abclonal, A24015), mouse anti-OCN (Takara, M188), rabbit anti-SOX9 (Abclonal, A19710), rabbit anti-Aggrecan (Abclonal, A11691),rabbit anti-β-actin (Abclonal, AC050), rabbit anti-DDB1 (Abclonal, A5022), rabbit anti-CUL4A (Abclonal, A2882), rabbit anti-CUL4B (Abclonal, A12696), rabbit anti-Ubiquitin (Proteintech,10201-2-AP), rabbit anti-Thoc6 (Proteintech, 20543-1-AP), rabbit anti-FLAG (Proteintech, 20543-1-AP). The following chemicals and reagents were used: Z-Guggulsterone (FXR inhibitor,HY-110066), Obeticholic acid (FXR agonist, HY-12222), Sodium carboxymethyl cellulose (HY-Y0703) and MG-132 (HY-13259) were purchased from MedChemExpress.

### Cell culture

All the cells were cultured at 37 °C in humidified incubators containing an atmosphere of 5% CO_2_. C3H10T1/2 cells were maintained in Minimum Essential Medium-α (α-MEM, Gibco, C12571500BT) containing 10% fetal bovine serum (FBS, Gibco,10099-141 C) and 1% Penicillin/Streptomycin (P/S) (Gibco, 15140-122).

### Isolation of mouse BMSCs, BMMs, osteoblasts cells, and chondrocytes

Femurs and tibias from 4-week-old mice were collected and flushed with phosphate-buffered saline (PBS) to obtain bone marrow cells. Nuclear cells were seeded at a density of 2 × 10^6^ cells per dish in 100-mm culture dishes. They were cultured in Minimum Essential Medium-α (α-MEM, Gibco, C12571500BT) supplemented with 10% FBS and 1% Penicillin/Streptomycin (P/S) (Gibco, 15140-122). After 24 h, adherent and non-adherent cells were harvested for subsequent BMSC and bone marrow-derived macrophage (BMM) culture. Primary osteoblasts cells were isolated from calvaria of 5-day-old neonates using 0.1% collagenase type I (Sigma-Aldrich, C0130), and were cultured in α-MEM, containing 10% FBS and 1% P/S. Primary chondrocytes cells were isolated from articular cartilage of 7-day-old neonates using 0.2% collagenase type II (Sigma-Aldrich, C2-BIOC) and were cultured in DMEM, containing 10% FBS and 1% P/S.

### In vitro osteoblastic differentiation and mineralization

To induce osteoblastic differentiation and mineralization, BMSCs or osteoblast were cultured in α-MEM containing 10% FBS, 50 μg/mL L-ascorbic acid (A4403, Sigma), and 1 080 mg/mL β-glycerophosphate (G9422, Sigma). 2 × 10^5^ BMSCs or osteoblasts were seeded in 12-well plates and cultured in osteogenic medium. ALP staining (Beyotime Institute of Biotechnology, P0321S) was performed on day 7, and Alizarin red S staining (Beyotime Institute of Biotechnology, C0148S) was performed on day 21. Alkaline phosphatase test kit (Beyotime Institute of Biotechnology, P0322S) was used to detect the ALP activity. For quantification of Alizarin red, the stain was washed off with 10% cetylpyridinium chloride (Solarbio, C9890) and measured using a spectrophotometer at 562-nm wavelength.

### In vitro osteoclastic differentiation

To induce osteoclastic differentiation, BMMs were seeded in 24-well plates supplemented with 20 ng/mL M-CSF (315-02, PeproTech, USA) for 3 days. Adherent cells identified as osteoclast progenitors, were further cultured in α-MEM containing 20 ng/mL M-CSF and 40 ng/mL RANKL (315-11 C, PeproTech, USA) for an additional 3–5 days.

### In vitro endochondral ossification assay

As described by Gretl et al. previously.^65^ BMSCs were seeded at a density of 20 000 cells/well in 12-well plates in chondrogenic DMEM/Ham’s F12 medium supplemented with 5% FBS, 10 μg/mL transferrin, 30 nmol/L sodium selenite, 0.2 mmol/L ascorbate-2-phosphate (Sigma, A8960) and 10 μg/mL insulin (Sigma, I9278). Cells were incubated in 6% O_2_ at 37 °C. At day 7, chondrogenic medium was replaced by osteogenic osteogenic medium and cells were cultivated in 21% O_2_ at 37 °C until day 10. RNA and protein were obtained at days 7 and 10 of cultivation.

### Plasmids

pOSE2-TA-Luc (D4243) and pRUNX2-TA-Luc (D4309) were purchased from Beyotime Biotechnology (Shanghai, China). FXR shRNA adenoviruses were prepared by OBiO (Shanghai, China). PGMLV-Mouse-FXR-3XFLAG-EF1-ZsGreen1-T2A-Puro (77973) and PGMLV-Mouse-EF1-ZsGreen1-T2A-Puro (10502) were prepared by Genomeditech (Shanghai, China). pECMV-RUNX2-FLAG (P6531) and pCMV-Thoc6-3XMyc-Neo (P55294) were prepared by MiaoLingBio, China. ΔAD, ΔRUNT, and ΔNLS were constructed into pcDNA3.1-FLAG Vector using full-length RUNX2 as a template. To construct different lengths of the Thoc6 promoter (Thoc6-luc (2 066), Thoc6-luc (574), fragments were amplified from genome DNA of C3H10T1/2 cells by PCR and then cloned into pGL3-Basic Vector (Promega, Madison, WI, USA). Point mutations in the Thoc6 promoter (Thoc6-luc (2 066 Mut)) were generated by site-specific mutagenesis using the overlap PCR extension method, with the longest Thoc6 promoter (Thoc6-luc (2 066)) serving as the template. The primers used are listed in Table S3.

### Protein half-life assay

Cells were treated with cycloheximide (MedChemExpress, HY-12320) for the indicated times, lysed, and subjected to western blotting.

### Quantitative real-time RT–PCR analysis

Cells were treated as indicated, and total RNA was isolated using TRIzol (T9424, Sigma-Aldrich) according to the manufacturer’s instructions. The isolated RNA was reverse transcribed using PrimeScript First Strand cDNA Synthesis Kit (TaKaRa, PR037A). The cDNA was mixed with ABISYR Green Master Mix (Applied Biosystems, Carlsbad, CA, USA) and subjected to amplification using a LightCycler 480 System (Roche, Switzerland). The primers used are listed in Table S2.

### Co-immunoprecipitation

Co-immunoprecipitation experiments were performed using the Co-immunoprecipitation Kit (Abbkine, KTI1020, Wuhan, China), following the manufacturer’s guidelines. Cells were seeded in 100 mm diameter dishes at 80%–90% confluence the day before transfection. Corresponding plasmids were transfected using SuperKine™ Lipo3.0 Efficient Transfection Reagent (Abbkine, BMU111-CN) according to the manufacturer’s instructions. After 48 h transfection, the cells were harvested and lysed in 1 mL of protein lysis buffer (20 mmol/L Tris-HCl (pH 7.4), 150 mmol/L NaCl, 1 mmol/L EDTA,1% Triton-X100) supplemented with protease inhibitor (11697498001, Roche, Switzerland). Whole-cell lysates were immunoprecipitated with corresponding primary antibodies and protein A/G magnetic beads at 4 °C overnight. The beads were washed three times with HEPES buffer and boiled for western blotting.

### Mass spectrometry

BMSCs from *FXR*^*fl/fl*^ and *FXR*^*prx1*^ mice littermates were subjected to osteogenic differentiation for 4 days. Binding proteins were co-immunoprecipitated with RUNX2 conjugated protein A/G magnetic beads, eluted with excessive amounts of elution buffer, and the eluates were subjected to mass spectrometry. LC–MS/MS for sequencing and data analysis was performed by JingJie PTM Biolab (Hangzhou, China) Co. Inc.

### Molecular docking

RUNX2–Thoc6 complex predictions were performed using Novopro’s methodology. Briefly, 3D models of RUNX2 and Thoc6 were generated using I-TASSER software. RUNX2 and Thoc6 docking was conducted in BIOVIA Discovery Studio Visualizer software using HDOCK algorithms. To obtain more accurate protein complex predictions, poses were reranked using the HRANK scoring program. Poses with high density, high HDOCK score, and low HRANK score were selected. The geometry of the selected docking solution was optimized using energy minimization with the Biovia Smart Minimizer algorithm. For the selected minimized solution, binding interface residues were identified, and interaction types (e.g. Hydrogen bonds, electrostatic, and hydrophobic interactions) were determined.^66^

### Cell viability assay

Cell proliferation was assessed using the CCK8 assay (MCE, HY-K0301) following the manufacturer’s instructions.

### Dual luciferase reporter assays

Cells were co-transfected with experimental reporter constructs. 48 h post-transfection, cells were lysed, and the activities of firefly luciferase and Renilla luciferase were analyzed according to the manufacturer’s instructions (Promega, Madison, WI. E1910). Each experiment was performed in triplicate using a multimode microplate reader (Tecan, infinite 200Pro, Switzerland). Results were expressed as the mean of triplicates ± standard deviation (SD).

### Analysis of bone formation rate by calcein labeling

Mice were injected intraperitoneally with 20 mg/kg calcein (Sigma, C0875-5G, 1 mg/mL in 2% NaHCO_3_ solution) 3 and 10 days prior to skeleton collection. The bones were fixed, dehydrated, and embedded in Embed-812 (Electron Microscopy Sciences). Samples were sectioned into 5 μm thick slices using a hard tissue cutter (RM2265, Leica, Wetzlar, Germany). Fluorescence-labeled images were captured using a microscope (BX51, Olympus). Histomorphometry analysis of the mineral apposition rate (MAR) and bone formation rate (BFR) was performed using ImageJ software.

### Micro-CT analysis

Femurs were harvested, and the surrounding soft tissue was removed. μCT analyses were conducted using a SkyScan 1174 μCT scanner. The scanning parameters were set to 65 kV and 150-μA. The resolution was 9 μm/pixel. Image reconstruction was performed using NRecon (version 1.6, SkyScan). Quantitative analysis to determine trabecular bone volume/total volume (BV/TV), trabecular thickness (Tb. Th), trabecular number (Tb. N), bone mass density (BMD), cortical thickness (Ct.Th), and trabecular separation (Tb. Sp) was conducted using CTAn (version 1.9, SkyScan). Coronal plane images of the femurs were generated using CTVol (version 2.0, SkyScan).

### Alcian blue and Alizarin red S staining

For Alcian blue and Alizarin red S staining of the skeleton, newborn mouse skins and organs were carefully removed using forceps. The remaining bodies were fixed in 95% ethanol for 1 day. After processing with acetone for 2 days to permeabilize cell membranes and dissolve fat, the cartilage was stained with Alcian blue 8GX (Solarbio, G8140) for 24 h. Subsequently, it was decolorized in 70% ethanol three times and 95% ethanol overnight. Following clearing in 1% KOH for 3 h, the bodies were immersed in Alizarin Red S (Solarbio, G8550) solution for 3–4 h to counterstain the bone. After clearing in 1% KOH in 20% glycerol solution, the skeletons were stored in 100% glycerol.

### Plasmid and short interfering RNA (siRNA) transfection

Cells seeded in plates were grown to 70%–80% confluence before plasmid or siRNA transfection. Plasmid transfection was performed using SuperKine™ Lipo3.0 Efficient Transfection Reagent (Abbkine, BMU111-CN) according to the manufacturer’s instructions. All siRNAs were purchased from genepharma (Shanghai, China). The sequences of the siRNAs used are as follows: siThoc6-1:GGGUCAGGCUGAGGUAUUUTT, siThoc6-2:GGGCAGAAAUUCUCAAGAATT.

### Hematoxylin and eosin (HE) staining, TRAP staining, immunohistochemistry, and immunofluorescence

Bone tissues were collected and fixed in 4% paraformaldehyde for 48 h. For newborn mice, bone tissues were decalcified in 0.5 mol/L EDTA (pH 7.26) (Solarbio, E1171) for 2 days. For postnatal mice, bone tissues were decalcified for 21 days. Subsequently, specimens were embedded in paraffin and sectioned at 5 μm. After deparaffinization and rehydration, bone sections were incubated in HE staining solution (Solarbio, G1120) and TRAP staining solution (Solarbio, G1492) according to the manufacturer’s instructions. For immunohistochemical staining, sections were de-waxed and rehydrated. Endogenous peroxidase. activity was blocked with a 3% H_2_O_2_ solution. Antigen retrieval was performed with protease K at 37 °C for 15 min. The antibody to OSX (Abcam, EPR21034, Rabbit monoclonal, 1:500) was added and incubated overnight at 4 °C. Anti-rabbit secondary antibody was added and incubated for 1 hour at room temperature, followed by color development with an ABC kit (ZSGB-Bio, SP-9000). For immunofluorescence, sections were blocked in PBS with 1% BSA (B2064, Sigma) for 1 h and then stained overnight with anti-FXR (R&D, PP-A9033A-00), OSX (Abcam, EPR21034, 1:500), anti-RUNX2 (Beyotime, AG1348,1:200), and anti-Thoc6 (Proteintech, 20543-1-AP, 1:200). Horseradish peroxidase-conjugated anti-rabbit IgG/ anti-mouse IgG/ anti-goat IgG (GK500710; Gene Tech, 1:300) were used as secondary antibodies at 37 °C for 30 min. DAPI (Sigma, D8417) was used for nuclear staining. Images were acquired with the Olympus FV1000 confocal microscope.

### ChIP assay

C3H10T1/2 cells were transfected with FXR cDNA. Chromatin immunoprecipitation (ChIP) was conducted using the Simple-ChIP Enzymatic Chromatin IP kit (Cell signaling technology, No. 9002) following the manufacturer’s protocol with slight modifications at 72 h post-transfection. Purified DNA served as a template for standard PCR and quantitative RT-PCR. The primer pairs employed are detailed in Table S2. Standard PCR products were subjected to electrophoresis on a 2% agarose gel and visualized under UV utilizing FluorChem E (ProteinSimple, California, USA). Quantitative RT-PCR results were interpreted in accordance with established protocols.

### ELISA analysis

Plasma samples were collected from mice and subjected to centrifugation at 5 000 revolutions per minute for 10 min at 4 °C. Subsequently, serum concentrations of PINP were quantified using the Mouse PINP EIA Kit (JL20174, Shanghai Future Industrial Limited by Share Ltd, China), following the manufacturer’s protocol.

### RNA-Seq

Total RNA was extracted from the BMSCs of *FXR*^*fl/fl*^ (*n* = 3) and *FXR*^*prx1*^ transgenic (*n* = 3) littermates following a 4-day osteogenic differentiation period using TRIzol Reagent. Library preparation was conducted by LC-Bio Technology Co, Ltd. Subsequent RNA sequencing was performed on an Illumina NovaSeq™ 6000 platform at LC-BioTechnology, Hangzhou, China, adhering to the manufacturer’s protocols.

### OVX model and FXR agonist treatment

Eighteen eight-week-old female C57BL/6 J mice were randomly assigned to three groups: a sham + vehicle group, an OVX+ vehicle group, and an OVX + OCA group. The sham + vehicle group received intragastric administration of 0.5% sodium carboxymethyl cellulose for four weeks without ovariectomy. The OVX+vehicle group underwent ovariectomy and subsequently received intragastric administration of 0.5% sodium carboxymethyl cellulose for four weeks. The OVX + OCA group underwent ovariectomy and then received intragastric administration of OCA (5 mg/kg/day) for four weeks. Eight weeks post-surgery, all mice were euthanized, and their bone tissues were collected for subsequent analysis.

### Statistical analysis

All data were presented as means ± standard deviation (SD) and analyzed using SPSS 13.0 statistical software (SPSS Inc., Chicago, IL, USA). To compare two groups, a two-tailed unpaired *t*-test was employed, while one-way ANOVA followed by Tukey’s post hoc test was used to assess differences among multiple groups. A *P*-value less than 0.05 was statistically significant.

## Supplementary information

Supplementary Materials

List of differential gene expression

## Data Availability

RNA-seq data and Mass spectrometry data have been deposited at the NCBI Sequence Read Archive at https://www.ncbi.nlm.nih.gov/sra with accession number PRJNA1096452. Other data in this study are available upon request from the corresponding author.
