# Supplementary material for: Nuclear farnesoid X receptor protects against bone loss by driving osteoblast differentiation through stabilizing RUNX2
Source: Bone Res. 2025 Jan 30;13:20. doi: 10.1038/s41413-024-00394-w (PMC11782663; doi:10.1038/s41413-024-00394-w)
Supplement: Supplementary file 1 — Supplementary Materials [file 41413_2024_394_MOESM1_ESM.docx]

**SUPPLEMENTAL MATERIAL FOR

Nuclear farnesoid X receptor protects against bone loss by driving osteoblast differentiation through stabilizing RUNX2**

Qi Dong^1,2*^，Haoyuan Fu^1,2*^，Wenxiao Li^1,2*^，Xinyu Ji^7^，Yingchao Yin^1,2^，Yiran Zhang^8^，Yanbo Zhu^6^，Guoqiang Li^1,2^，Huiyang Jia^1,2^，Heng Zhang^1,2^，Haofei Wang^1,2^，Jinglue Hu^2^，Ganggang Wang^5^，Zhihao Wu^4^，Yingze Zhang^1,2^，Sujuan Xu^1,2,3#，^Zhiyong Hou^1,2#^
^1^Department of Orthopedic Surgery，Third Hospital of Hebei Medical University，Shijiazhuang，Hebei，China. ^2^Orthopaedic Research Institute of Hebei Province，Third Hospital of Hebei Medical University，Shijiazhuang，Hebei，China.
^3^ Hebei Key Laboratory for Diabetic Kidney Disease，Third Hospital of Hebei Medical University，Shijiazhuang，Hebei，China.
^4^School of Preclinical Medicine，Wannan Medical College，Wuhu，Anhui，China.
^5^Pudong Hospital，Fudan University，Shanghai，China.
^6^Hebei Food Safety Key Laboratory，Key Laboratory of Special Food Supervision Technology for State Market Regulation，Hebei Engineering Research Center for Special Food Safety and Health，Hebei Food Inspection and Research Institute，Shijiazhuang，Hebei，China.
^7^Department of Cardiology，Third Hospital of Hebei Medical University，Shijiazhuang，Hebei，China.
^8^School of Medicine，Nankai University，Tianjin，China.
*Qi Dong，Haoyuan Fu，Wenxiao Li contributed equally to this work.
^#^Corresponding author: Dr. Zhiyong Hou，Address: Department of Orthopaedical Surgery，the Third Hospital of Hebei Medical University，Shijiazhuang 050051，China. Mail address: [drzyhou@hebmu.edu.cn](mailto:drzyhou@hebmu.edu.cn)
Dr. Sujuan Xu，Address: Hebei Key Laboratory for Diabetic Kidney Disease，the Third Hospital of Hebei Medical University，Shijiazhuang 050051，China. Mail address: sujuanxu@hebmu.edu.cn **Supplementary Figure.1**
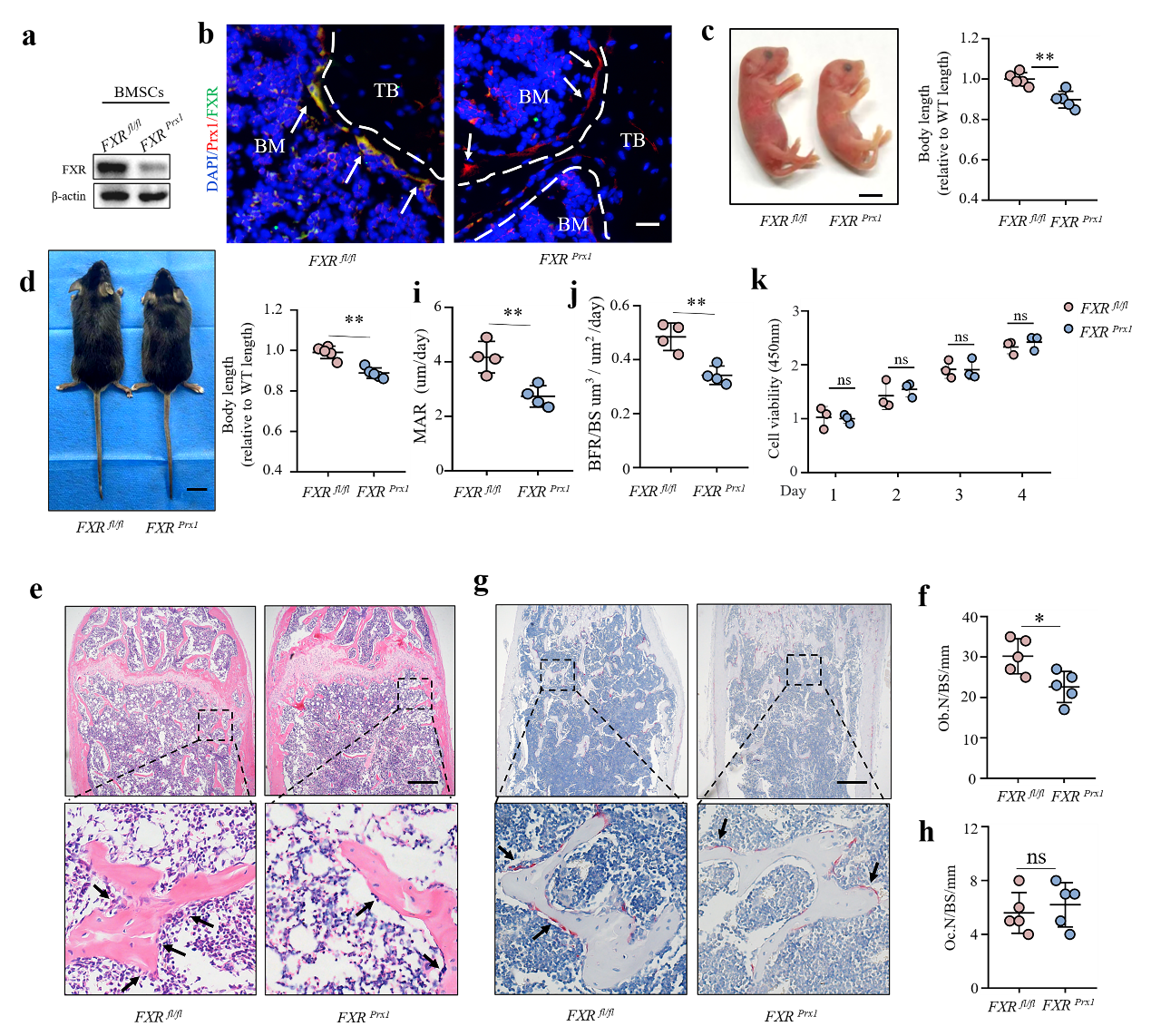

**Supplementary Figure.1 a** Western blot analysis of FXR in BMSCs from 4-week-old male FXR*^fl/fl^* and FXR*^Prxl^* mice cultured in osteogenic medium for 7 days. **b** Immunofluorescence assay for FXR expression in MSCs (Prx1-a marker of MSCs) of FXR*^fl/fl^* and FXR*^Prx1^* mice. n = 3. Scale bars, 50 μm. BM means bone marrow. TB means trabecular bone. **c** Representative view and quantification of FXR*^fl/fl^* and FXR*^Prx1^* newborn mice. n=5. Scale bar=5 mm. **d** Representative views and quantification of 8-week-old male FXR*^fl/fl^* and FXR*^Prx1^* mice. n=5. Scale bar=1 cm. **e，f** Representative images of hematoxylin and eosin (HE) staining (e) and quantitative analysis of osteoblasts in the femurs area from FXR*^fl/fl^* and FXR*^Prxl^* mice. n=5. Scale bar=500 μm. **g，h** TRAP staining (g) and quantification (h) of femur area from FXR*^fl/fl^* and FXR*^Prxl^* mice. n=5. **i，j** Quantitative analysis of mineralization apposition rate (MAR) and bone formation rate (BFR) from 8-week-old male FXR*^fl/fl^* and FXR*^Prx1^* mice. n = 4. **k** BMSCs viability was measured using the CCK8 assay. n=3. *P < 0.05， **P < 0.01，NS，no significant difference. Data are represented as mean ± SEM. **Supplementary Figure.2**

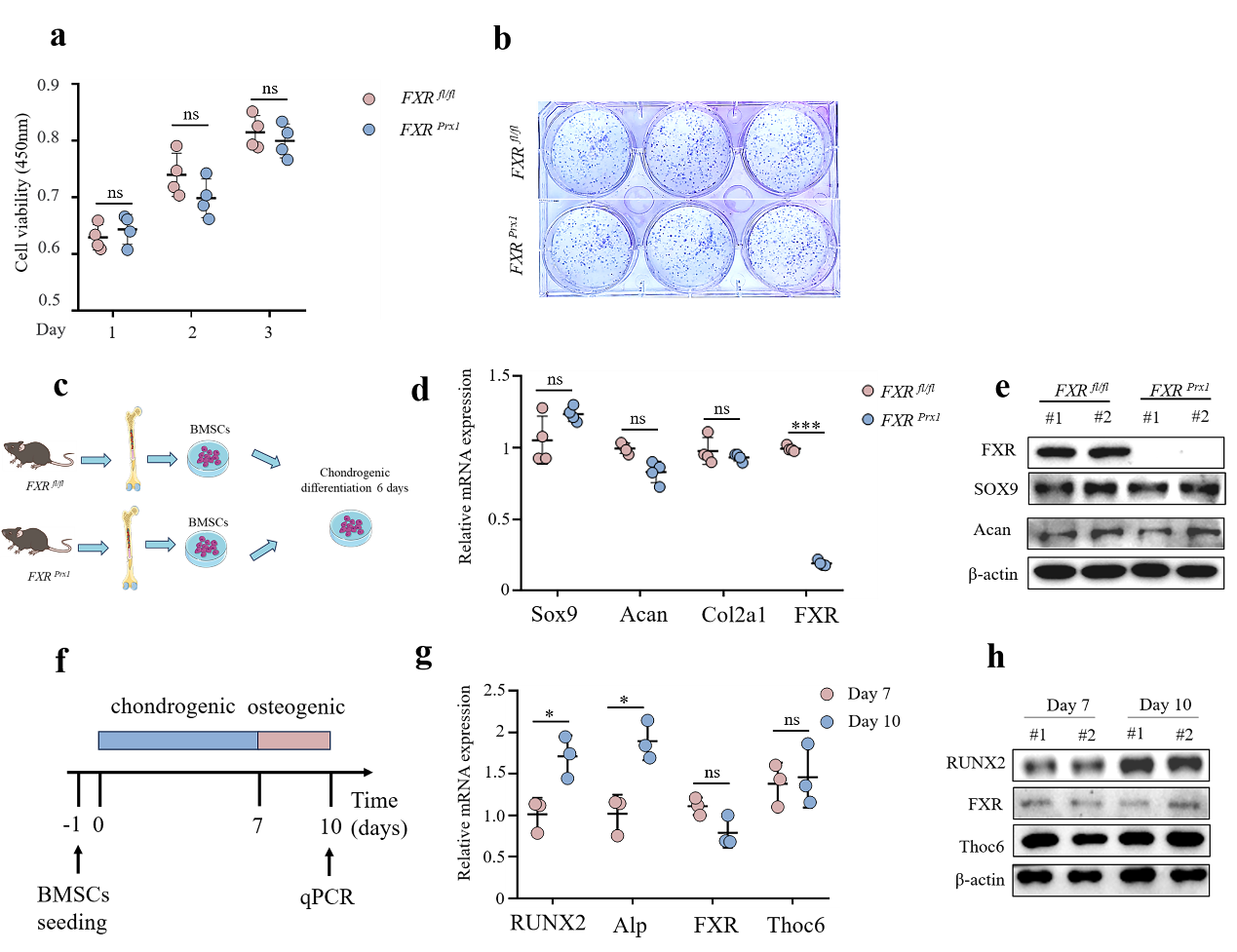
 **Supplementary Figure.2
a** Chondrocytes viability was measured using the CCK8 assay. n=4. **b** Representative image of crystal violet staining of cell proliferation assay. n=3. **c** Schematic graph of the strategy to induce chondrocytes differentiation. **d** RT-PCR analysis was performed to examine relative mRNA levels of *Sox9， Acan， Col2α1，and FXR* in chondrocytes from FXR^fl/fl^ and FXR^Prxl^ mice. n=4.  **e** Western blot analysis was performed to examine *FXR*，*Sox9*，and *Acan* in chondrocytes from FXR*^fl/fl^* and FXR*^Prxl^* mice. **f** Schematic graph of the in vitro endochondral ossification assay. **g** RT-PCR analysis was performed to examine relative mRNA levels of *RUNX2，Alp，FXR and Thoc6* in differentiated cells. Samples were collected at 7 and 10 days after differentiation*.* n=3. **h** Western blot analysis was performed to examine *RUNX2*，*FXR*，and *Thoc6* in differentiated cells. Samples were collected at 7 and 10 days after differentiation*.* *P < 0.05，***P < 0.001，NS，no significant difference. Data are represented as mean ± SEM.


 **Supplementary Figure.3**

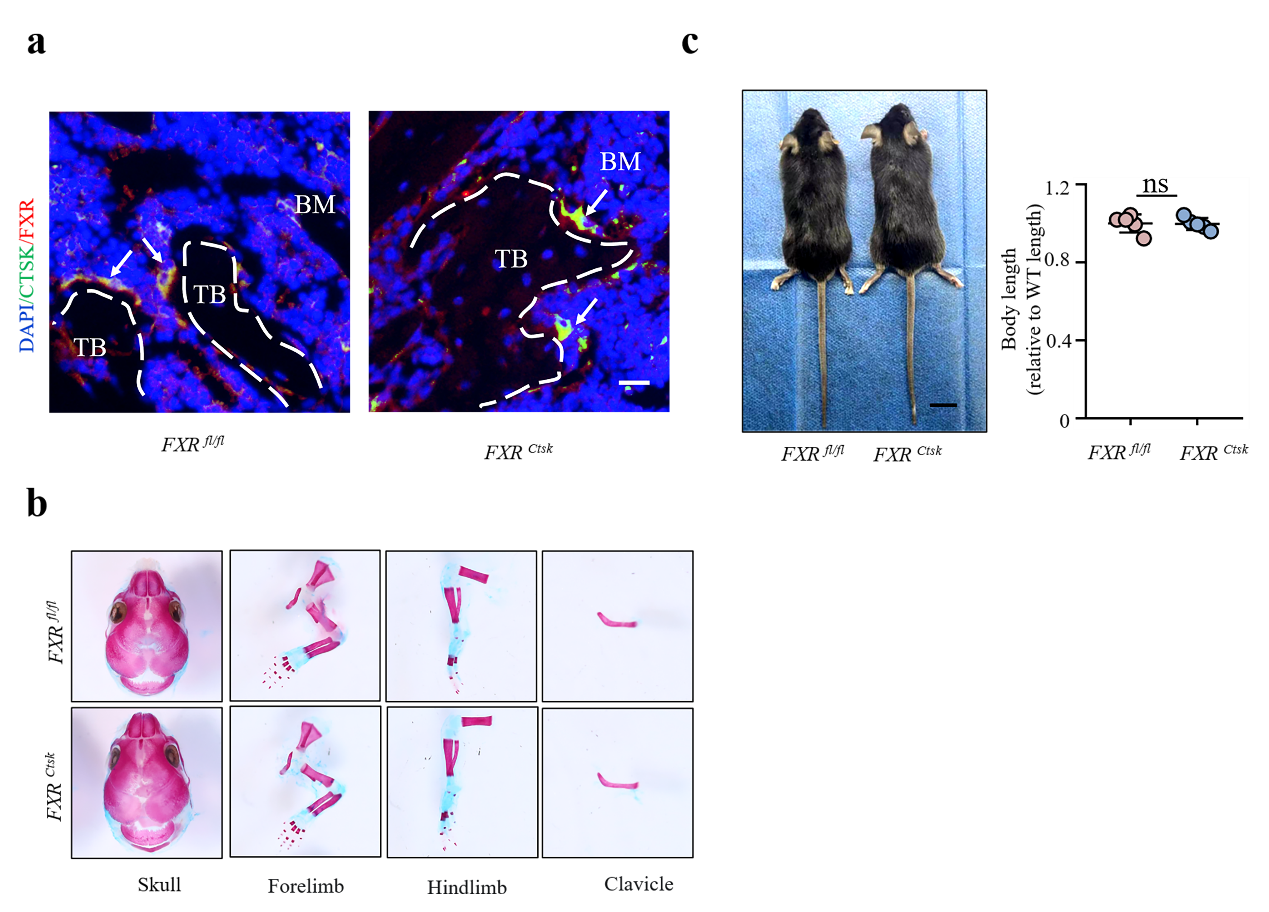
 **Supplementary Figure.3 a** Immunofluorescence assay for FXR expression in osteoclast (CTSK-a marker of osteoclast) of FXR*^fl/fl^* and FXR*^Ctsk^* mice. n = 3 per group. Scale bars，50 μm. BM means bone marrow. TB means trabecular bone. **b** Representative double-stained with alcian blue and alizarin red S image of skeletal preparations from FXR*^fl/fl^* and FXR*^Ctsk^* newborns. **c** Representative view and quantitative analysis of 8-week-old male FXR*^fl/fl^* and FXR*^Ctsk^* mice. n=5. Scale bar=1 cm. NS，no significant difference. Data are represented as mean ± SEM.

 **Supplementary Figure.4**

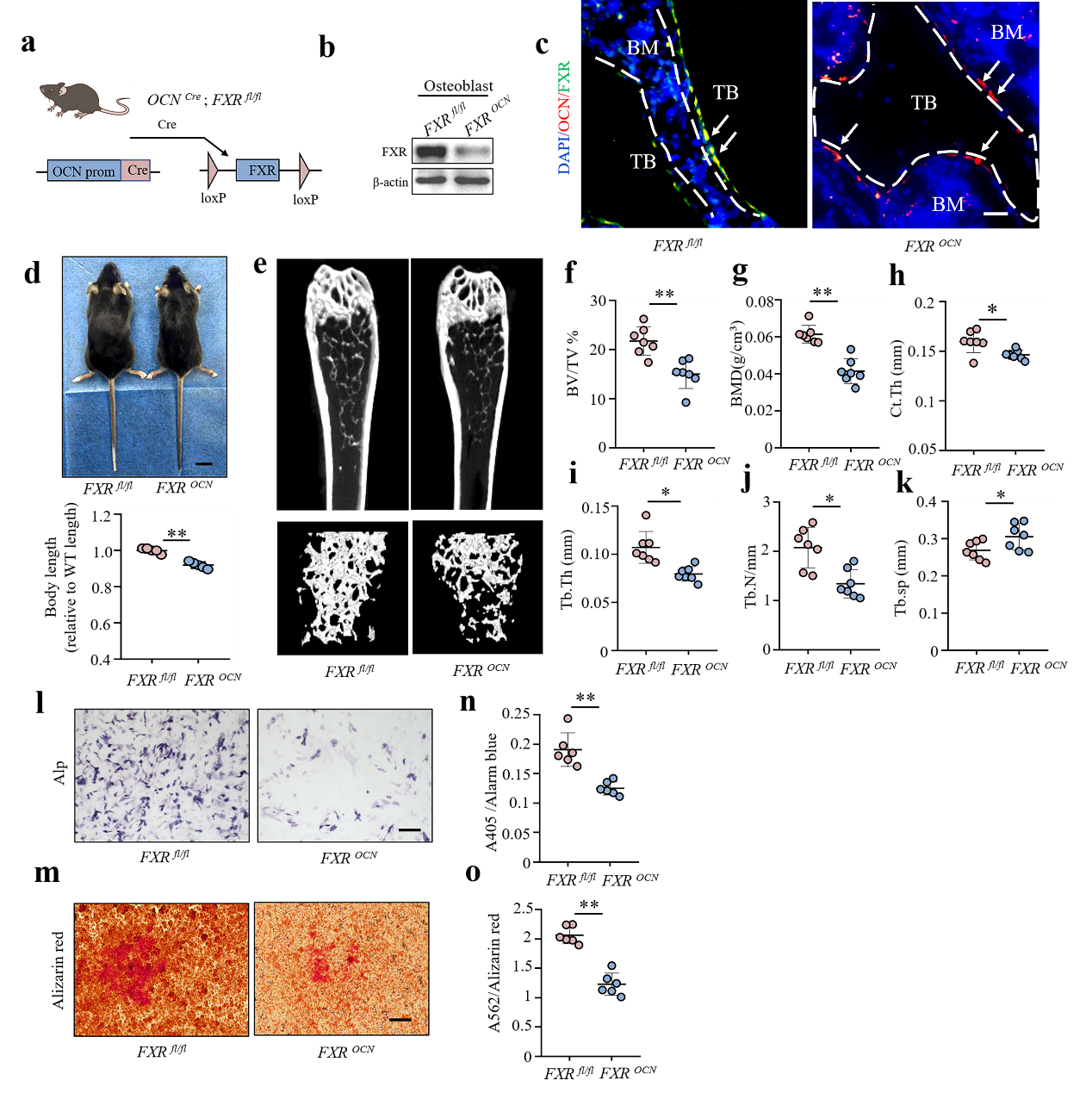
 **Supplementary Figure.4 Inactivation of FXR in osteoblast leads to impaired bone ossification**.
**a** Illustration of osteoblast lineage-specific FXR knockout mice, which were generated via the crossbreeding of FXR*^fl/fl^* mice with OCN-Cre transgenic mice. **b** Western blot analysis of FXR in calvarial cells from 4-week-old male FXR*^fl/fl^* and FXR*^OCN^* mice cultured in osteogenic medium for 7 days. **c** Immunofluorescence assay for FXR expression in osteoblast (OCN-a marker of osteoblast) of FXR*^fl/fl^* and FXR*^OCN^* mice. n=3. Scale bars, 50 μm. BM means bone marrow. TB means trabecular bone. **d** Representative views quantification of 8-week-old male FXR*^fl/fl^* and FXR*^OCN^* mice. **e** Representative micro-CT images of whole femoral (top) and trabecular (bottom) bones from 8-week-old male FXR*^fl/fl^* and FXR*^OCN^* mice. **f-k** Representative micro-CT images of distal femurs in 8-week-old FXR*^fl/fl^* and FXR*^OCN^* mice with morphometric analysis of bone volume per tissue volume (BV/TV)，bone mass density (BMD)，trabecular thickness (Tb.Th)，trabecular number (Tb.N)，trabecular spacing (Tb.Sp) and cortical thickness (Ct.Th). n = 7. **l，m** Representative images of Alp staining and Alizarin red staining of calvarial cells from FXR*^fl/fl^* and FXR*^OCN^* mice after cultured in osteogenic medium. **n，o** Quantitative analysis of Alp activity and Alizarin red staining. n = 6. *P < 0.05，**P < 0.01. Data are represented as mean ± SEM.
 **Supplementary Figure.5**
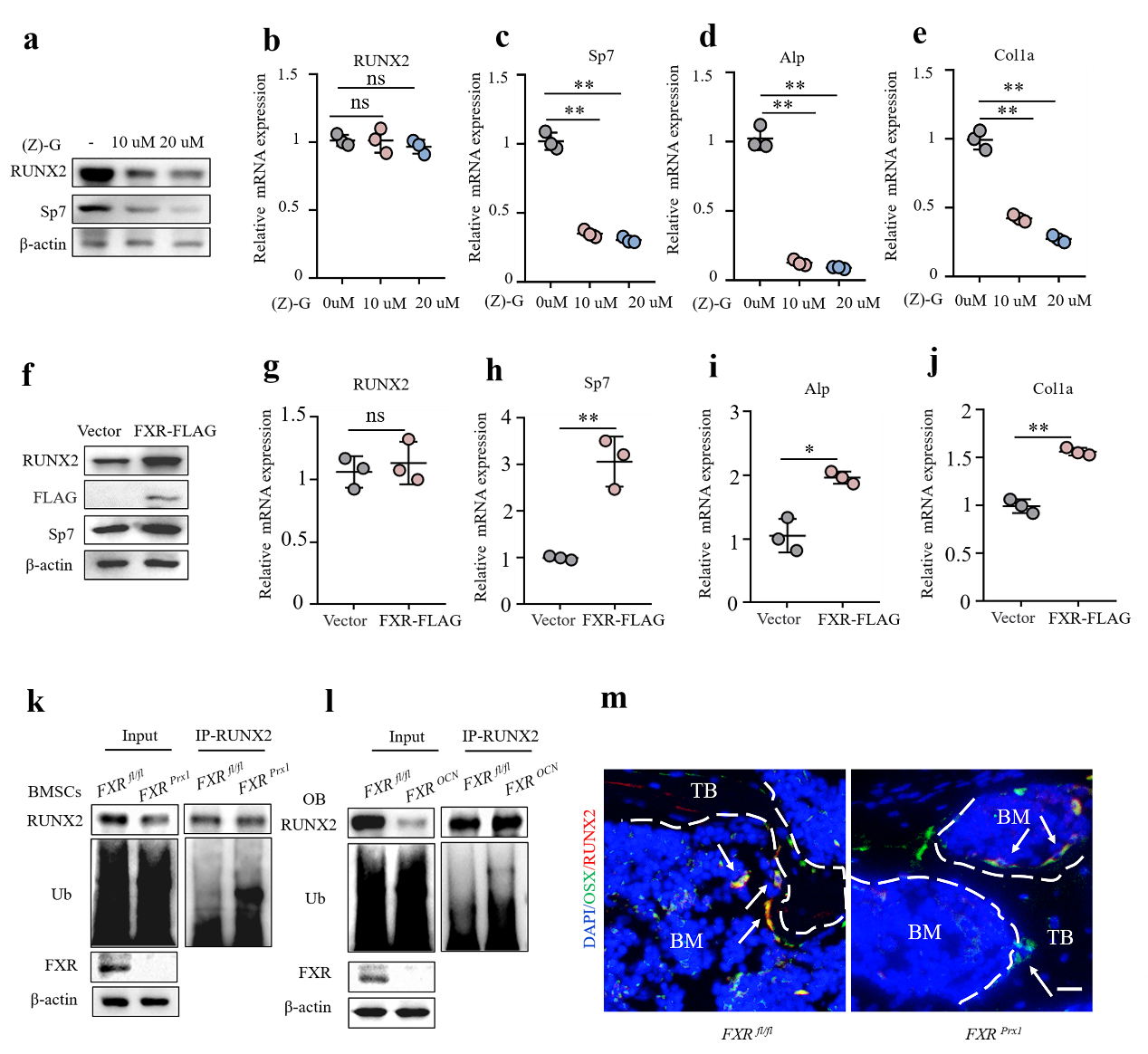
 **Supplementary Figure.5
a** Western blot analysis of RUNX2，Sp7 protein levels in BMSCs after 4 days of osteoblastic differentiation treated with or without FXR inhibitor (Z)-G (Z-Guggulsterone). **b-e** Quantitative RT-PCR analysis of *RUNX2*，*Sp7*，*Alp*，*Col1a* genes expression in BMSCs after 4 days of osteoblastic differentiation treated with or without FXR inhibitor (Z)-G. n=3. **f** Western blot analysis of FXR， RUNX2，Sp7 in BMSCs after 4 days of osteoblastic differentiation transfection with or without FXR-FLAG. **g-j** Quantitative RT-PCR analysis of *RUNX2*，*Sp7*，*Alp*，*Col1a* genes expression in BMSCs after 4 days of osteoblastic differentiation transfection with or without FXR-FLAG. n=3. **k** Immunoblot of RUNX2 polyubiquitination levels in BMSCs from FXR*^fl/fl^* and FXR*^prx1^* mice. **l** Immunoblot of RUNX2 polyubiquitination levels in osteoblast from FXR*^fl/fl^* and FXR*^OCN^* mice. **m** Immunofluorescence assay for RUNX2 expression in osteoblast (OSX-a marker of osteoblast) of FXR*^fl/fl^* and FXR*^prx1^* mice. n = 3. Scale bars, 50 μm. BM means bone marrow. TB means trabecular bone. *P < 0.05，**P < 0.01，NS，no significant difference. Data are represented as mean ± SEM.
 **Supplementary Figure.6**
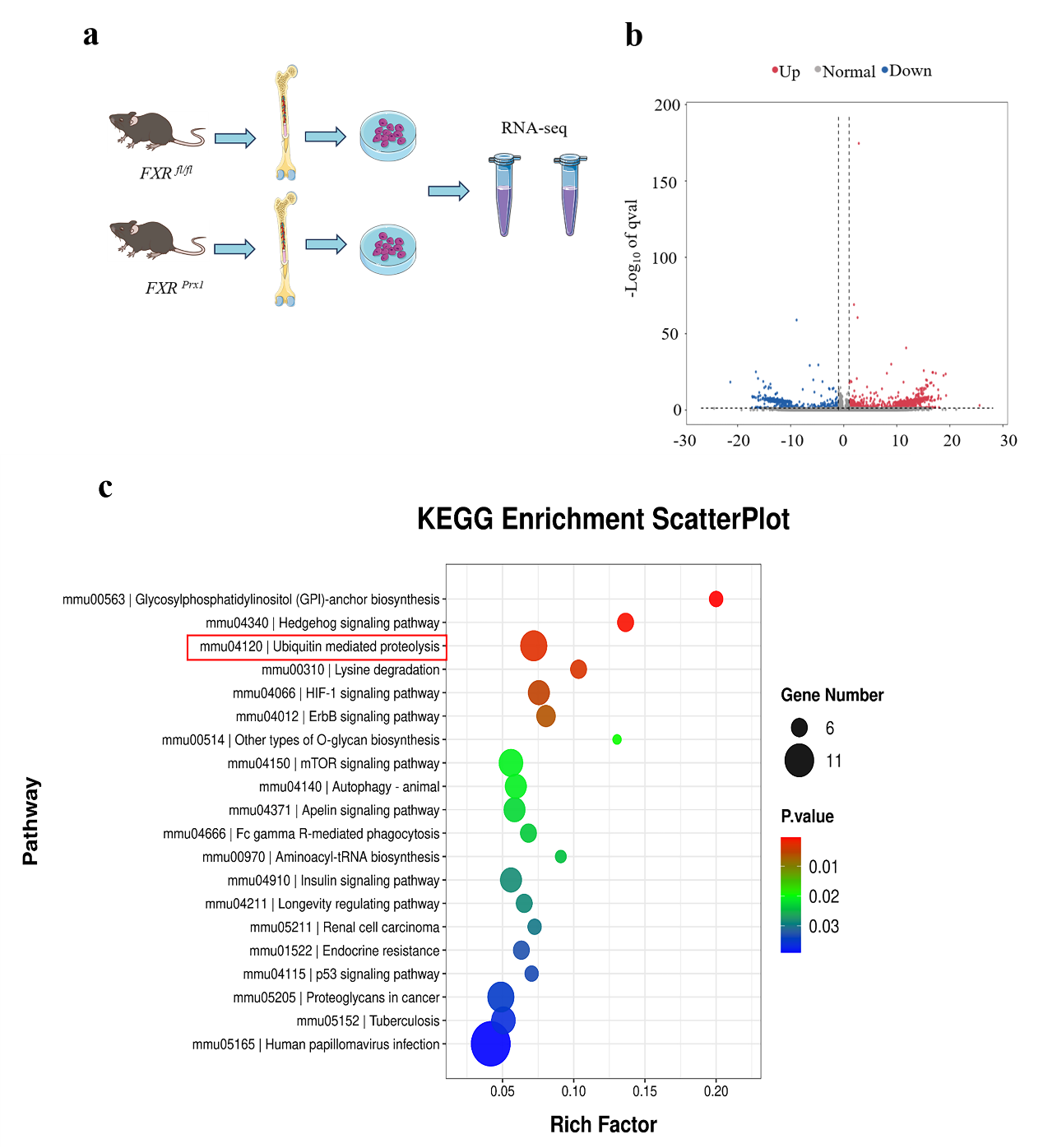
 **Supplementary Figure.6 a** Schematic graph of the RNA-seq strategy to identify genes which were regulated by FXR. Total RNA was isolated from BMSCs，which was cultured from 4-week male FXR*^fl/fl^* and FXR*^prx1^* mice，after 4 days of osteoblastic differentiation followed by RNA sequencing analysis. Kolmogorov–Smirnov (K–S) test was used for testing the correlation between the FXR*^fl/fl^* and FXR*^prx1^* set. **b** Volcano plot displays global gene expression in BMSCs which was cultured from 4-week male FXR*^fl/fl^* and FXR*^prx1^* mice. **c** Kyoto encyclopedia of genes and genomes (KEGG) pathway enrichment analysis of differentially expressed genes in BMSCs，which was cultured from 4-week male FXR*^fl/fl^* and FXR*^prx1^* mice.
 **Supplementary Figure.7**


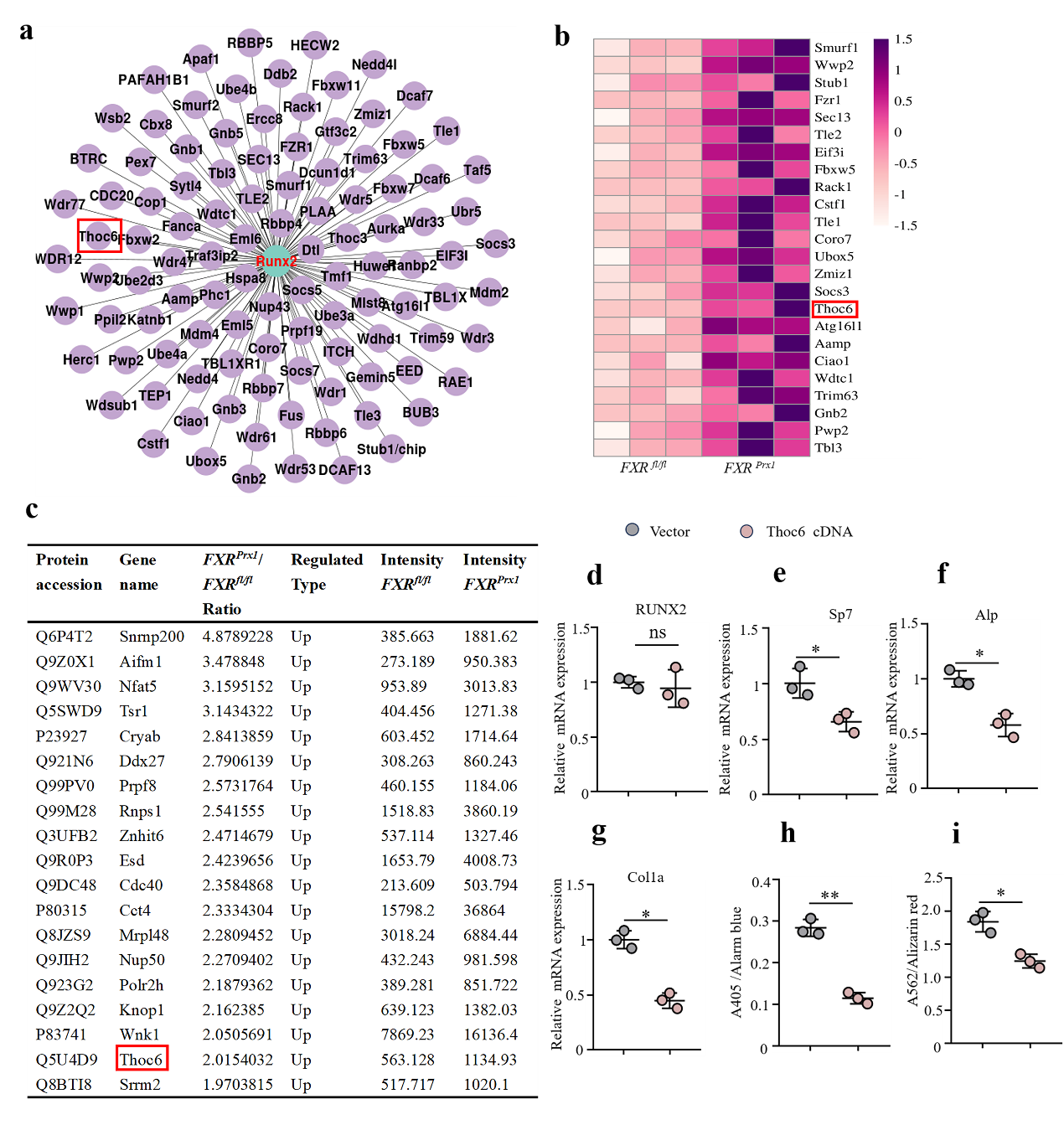
 **Supplementary Figure.7 a** The potential E3 ligases of RUNX2 as a substrate were predicted using Ubibrowser. **b** RNA-sequence analysis of up-regulated gene profiles of the predicted E3 ligases might interact with RUNX2. **c** Mass spectrometry analysis of the intensity of counted peptides. **d-g** Quantitative RT-PCR analysis of *RUNX2*，*Sp7*，*Alp*，*Col1a* genes expression in BMSCs after 4 days of osteoblastic differentiation transfection with or without Thoc6 cDNA. n=3. **h，i** Quantification analysis of Alp activity (h) and Alizarin red staining (i) of BMSCs from FXR*^fl/fl^* and FXR*^prx1^* mice cultured in osteogenic medium. n = 3. *P < 0.05，**P < 0.01，NS，no significant difference. Data are represented as mean ± SEM.
**Supplementary Figure.8**

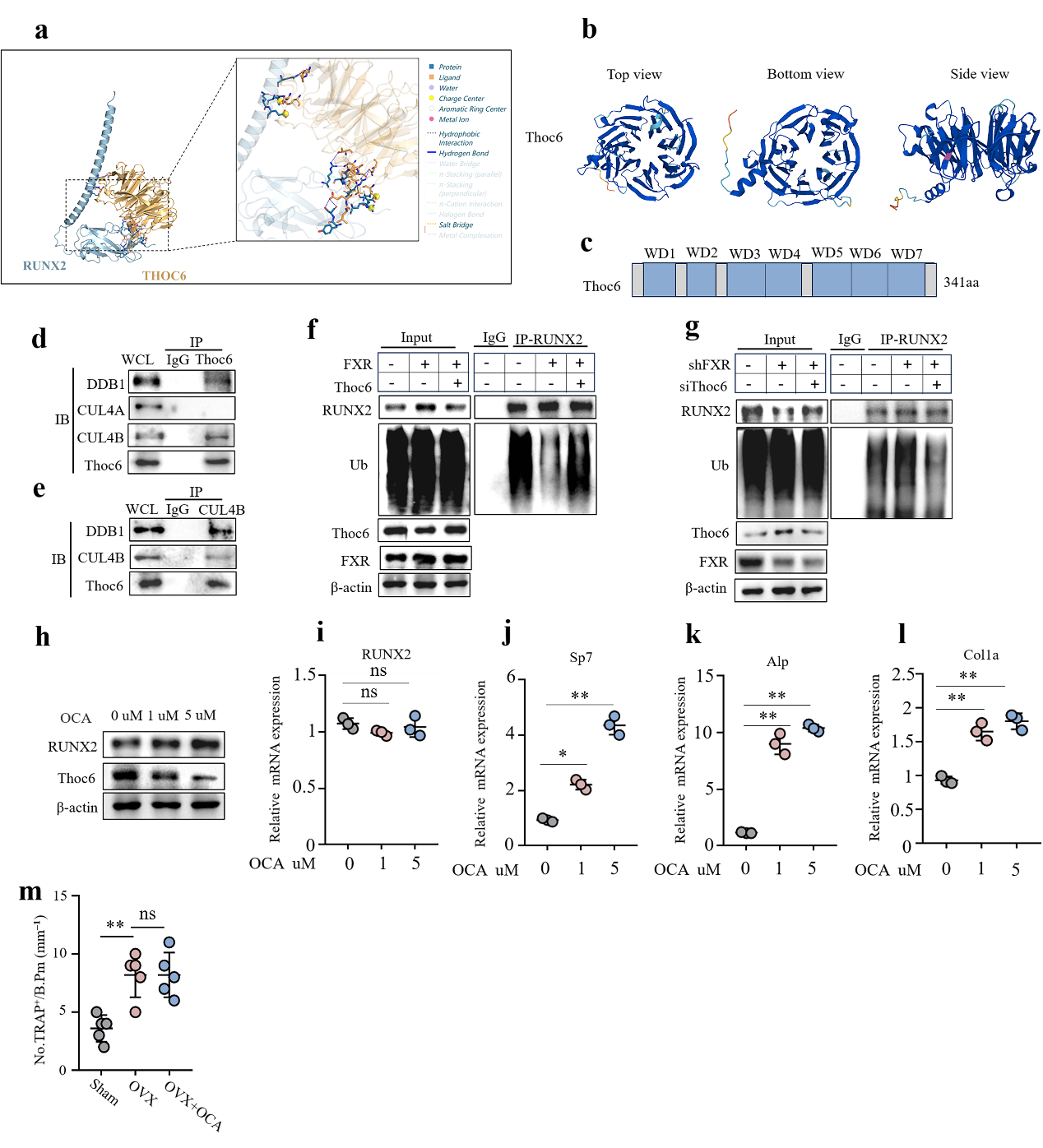


**Supplementary Figure.8
a** Magnified view of the key residues for interaction between mouse RUNX2 and Thoc6. **b** Structure of the Thoc6 β-Prp as viewed from the top，side，and bottom perspectives. **c** Schematic graph that reflects the domain and features of Thoc6. **d，e** Interaction between THOC6 and CUL4B in BMSCs. BMSCs were lysed after 4 days of osteoblastic differentiation，immunoprecipitated with anti-IgG control，anti-Thoc6 (d) or anti-CUL4B (e) antibody and protein A/G magnetic beads，and immunoblotted with the indicated antibodies. WCL means whole cell lysate. **f** After C3H10T1/2 cells were transfected with FXR cDNA or/and Thoc6 cDNA for 48 hours，lysed，immunoprecipitated with anti-RUNX2 conjugated protein A/G magnetic beads，and immunoblotted with the indicated antibodies. **g** After C3H10T1/2 cells were transfected with FXR shRNA or/and Thoc6 shRNA for 48 hours，lysed，immunoprecipitated with anti-RUNX2 conjugated protein A/G magnetic beads，and immunoblotted with the indicated antibodies. **h** Western blot analysis of RUNX2, Thoc6 expression in BMSCs after 4 days of osteoblastic differentiation treated with or without OCA. (OCA-an agonist of FXR). **i-l** Quantitative RT-PCR analysis of *RUNX2*， *Sp7*，*Alp*， *Col1a* genes mRNA levels in BMSCs after 4 days of osteoblastic differentiation treated with or without OCA. n=3. **m** TRAP staining quantification of femur area from sham，OVX，OVX treated with OCA groups. n = 6. *P < 0.05， **P < 0.01，NS，no significant difference. Data are represented as mean ± SEM.
 **Supplementary Figure.9**
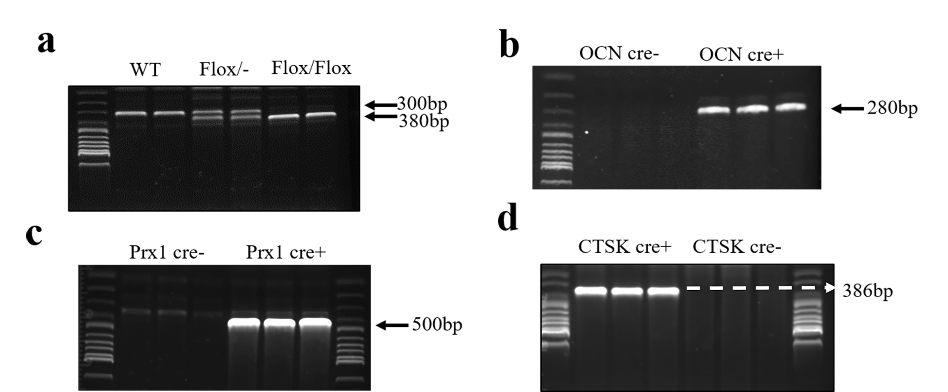


**Supplementary Figure.9**
**a-d** Genotype validation of bone mesenchymal stem cells–farnesoid X receptor–knockout (BMSC-FXR-KO)，osteoclast–FXR-KO mice and osteoblast–FXR-KO mice. (a) The 300-bp and the 380-bp bands represent the wild-type (WT) and the FXR*^fl/fl^* alleles respectively. (b-d) The 500-bp，386-bp and 280-bp bands represent the Prx1-cre，Ctsk-cre and OCN-cre transgenes that present in the specific KO mice. The Prx1 promoter drives Cre expression mainly in mesenchymal stem cells. The Ctsk promoter drives Cre expression in the osteoclast. The OCN promoter drives Cre expression in the osteoblast.

| **Supplementary Table S1. Primers used in mouse identification of genotyping.** | | | | | | |
| --- | --- | --- | --- | --- | --- | --- |
| **Gene name** | **Forward (5’-3’)** | | **Reverse (5’-3’)** | | **Products length (bp)** | |
| FXR flox genotyping | ATAGACAACCCCAGTGACCC | | TCTAAAGGATAGCCGAATCT | | 300(WT) 380(flox) | |
| Prx1-cre genotyping | GGGTCTGTAAAACGTCAGGC | | GTGAAACAGCATTGCTGTCACTT | | 0(WT) 500(Targeted) | |
| Ctsk-cre genotyping | GTGAAACAGCATTGCTGTCACTT | | GAGCCCAGATCCACATCTGAACTG | | 0(WT) 386(Targeted) | |
| OCN-cre genotyping | CAAATAGCCCTGGCAGATTC | | TGATACAAGGGACATCTTCC | | 0(WT) 280(Targeted) | |
|  |  | |  | |  | |
|  |  | |  | |  | |
|  |  | |  | |  | |
|  |  | |  | |  | |
|  |  | |  | |  | |
| **Supplementary Table S2. Primers used in qPCR.** | | | | | |  |
| **Gene name** | **Forward (5’-3’)** | **Reverse (5’-3’)** | | **Application** | |  |
| β-actin | TCTGCTGGAAGGTGGACAGT | CCTCTATGCCAACACAGTGC | | qPCR | |  |
| RUNX2 | CATTTGCACTGGGTCACACGTA | GAATCTGGCCATGTTTGTGCTC | | qPCR | |  |
| Osterix | ACTCATCCCTATGGCTCGTG | GGTAGGGAGCTGGGTTAAGG | | qPCR | |  |
| FXR | TGGGTACCAGGGAGAGACTG | CGGAAGAAACCTTTGCAGCC | | qPCR | |  |
| ALP | CCAACTCTTTTGTGCCAGAGA | GGCTACATTGGTGTTGAGCTTTT | | qPCR | |  |
| Col1a | GCTCCTCTTAGGGGCCACT | CCACGTCTCACCATTGGGG | | qPCR | |  |
| Thoc6 | GCTGGCGATGGAGAGGTCAAG | CAAGGCATTGATTTCGGGTACTTCC | | qPCR | |  |
| Thoc6 | ATCTGTCCACCTGAGTCTACAG | TGGGGCGATTCAGGA | | ChIP-qPCR | |  |

| **Supplementary Table S3. Primers used in cloning.** | | |
| --- | --- | --- |
| **Gene nAme** | **ForwArd (5’-3’)** | **Reverse (5’-3’)** |
| ΔAD | TAGTCCAGTGTGGTGGAATTCGCCACCATGACCATGGTGGAGATCATCG | ATGGTCTTTGTAGTCCTCGAGATATGGCCGCCAAACAGAC |
| ΔRUNT | TAGTCCAGTGTGGTGGAATTCGCCACCATGAGGCACAGACAGAAGCTTG | ATGGTCTTTGTAGTCCTCGAGATATGGCCGCCAAACAGAC |
| ΔNLS | TAGTCCAGTGTGGTGGAATTCGCCACCATGCGCATTCCTCATCCCAGTATG | ATGGTCTTTGTAGTCCTCGAGATATGGCCGCCAAACAGAC |
| Thoc6-luc(2066) | GGTACCTGGTGATTCACCTCCAAGGAC | CTCGAGCTGTCCCGCCTTCTAACCTC |
| Thoc6-luc(574) | GGTACCATCTGTCCACCTGAGTCTACAG | CTCGAGCTGTCCCGCCTTCTAACCTC |
| Thoc6-luc(2066) Mutation | AGGCTATACACCAACCGCCTTGG | CCAAGGCGGTTGGTGTATAGCCT |
|  |  |  |
|  |  |  |
|  |  |  |
